# Proteases from *Entamoeba* spp. and Pathogenic Free-Living Amoebae as Virulence Factors

**DOI:** 10.1155/2013/890603

**Published:** 2013-02-07

**Authors:** Jesús Serrano-Luna, Carolina Piña-Vázquez, Magda Reyes-López, Guillermo Ortiz-Estrada, Mireya de la Garza

**Affiliations:** Departamento de Biología Celular, Centro de Investigación y de Estudios Avanzados del Instituto Politécnico Nacional, Avenida Instituto Politécnico Nacional 2508, 07360 México, DF, Mexico

## Abstract

The standard reference for pathogenic and nonpathogenic amoebae is the human parasite *Entamoeba histolytica*; a direct correlation between virulence and protease expression has been demonstrated for this amoeba. Traditionally, proteases are considered virulence factors, including those that produce cytopathic effects in the host or that have been implicated in manipulating the immune response. Here, we expand the scope to other amoebae, including less-pathogenic *Entamoeba* species and highly pathogenic free-living amoebae. In this paper, proteases that affect mucin, extracellular matrix, immune system components, and diverse tissues and cells are included, based on studies in amoebic cultures and animal models. We also include proteases used by amoebae to degrade iron-containing proteins because iron scavenger capacity is currently considered a virulence factor for pathogens. In addition, proteases that have a role in adhesion and encystation, which are essential for establishing and transmitting infection, are discussed. The study of proteases and their specific inhibitors is relevant to the search for new therapeutic targets and to increase the power of drugs used to treat the diseases caused by these complex microorganisms.

## 1. Introduction

Amoeba is a general name that is used for protists that form a large and diverse assemblage of eukaryotes that are characterized by various types of pseudopodia [1, 2]. Some amoebae are pathogenic and even parasitic to human and other vertebrate hosts. The four amoebae that are dealt with in this paper have been classified under two Super Groups, Amoebozoa and Excavata, as follows: (a) *Entamoeba, Acanthamoeba,* and* Balamuthia* are classified under the Super Group Amoebozoa; (b) *Naegleria fowleri* is classified under Super Group Excavata [1, 2]. The genus *Entamoeba* includes several species, such as* E. histolytica*, which causes amoebiasis, an infection in the gut characterized by invasion of the intestinal mucosa that occasionally spreads to other organs, mainly the liver, and *E*. *dispar* and *E. moshkovskii*, which are morphologically similar to *E. histolytica *and have been recently recognized as separate species. Although *E. dispar *and *E. moshkovskii* have no apparent invasive potential, they exhibit some pathogenicity [3, 4]. Molecular phylogeny analysis places the genus *Entamoeba* on one of the lowermost branches of the eukaryotic tree, closest to *Dictyostelium *[5]. Although *Entamoeba*, originally thought to lack mitochondria, nuclear-encoded mitochondrial genes and a remnant organelle have now been identified. The establishment of *E. dispar* and* E*. *moshkovskii *as distinct, but closely related, species has profound implications for the epidemiology of amoebiasis because most asymptomatic infections are now attributed to these noninvasive amoebae [3].

 Free-living amoebae belonging to the genera *Acanthamoeba*, *Balamuthia*, and *Naegleria *are responsible for opportunistic and nonopportunistic infections in humans and other animals. The distinction between parasitic and free-living protozoa is generally sharp, with organisms falling readily into one or the other category. Some of the free-living amoebae are unusual in that they straddle the line separating the two groups of organisms and yet are as destructive as any of the classic parasitic protozoa [52]. Unlike their parasitic counterparts, these free-living amoebae are not well adapted for parasitism. Furthermore, as free-living forms with a broad distribution, they are not dependent upon a host for transmission and spread. Unlike the parasite *E. histolytica*, free-living amoebae are mitochondrion-bearing aerobic organisms that can complete their respective life cycles in the environment without a host; thus, these organisms have been called amphizoic amoebae in recognition of their ability to live endozoically, although they are capable of free-living existence [52].

 All pathogenic amoeba species have in common the capability to phagocytose bacteria, erythrocytes, and cell detritus. The major virulence factors are adhesins, toxins, amoebapores, and proteases, which lead to the lysis, death, and destruction of a variety of cells and tissues in the host. Because of their relevance to amoebic pathogenesis, proteolytic enzymes are of particular interest. Proteases are important in tissue invasion, migration, and host pathology. The main goal of this paper is to review the proteases of the *Entamoeba* genus and of free-living amoebae.

## 2. *Entamoeba histolytica*

Amoebiasis is a human parasitic infection caused by *Entamoeba histolytica*,  an extracellular protozoan.  Cysts are transmitted through the fecal-oral route by contaminated water or food. Parasite destruction of host tissues appears to be the basis of amoebiasis, which leads to invasive disease pathologies such as intestinal amoebiasis, which is mainly characterized by lesions in the colon that produce fever, abdominal pain, dysentery, and ulcerative colitis with mucous and blood. Amoebae can spread through the portal vein to other organs such as the liver, lungs, kidneys, and brain. Hepatic amoebiasis is characterized by liver abscesses that can be fatal [53–57].

 Amoebiasis is the third leading cause of death due to parasites, after malaria and schistosomiasis. Amoebiasis presents a high index of morbidity and mortality, mainly in developing countries. According to the World Health Organization (WHO), 500 million people are infected with amoebae; 10% of infected individuals have virulent *E. histolytica*, resulting in 40,000–100,000 deaths annually [56]. *E. histolytica *infects only humans. The life cycle of *E*. *histolytica *consists of two main stages: the trophozoite (also called amoeba) or invasive form, and the cyst or infective form. Cysts can tolerate the stomach acidic pH and excyst in the terminal ileum. Trophozoites colonize the large intestine, and in later phases of amoebiasis, they invade the epithelium and mucosa. In response to unknown stimuli, amoebae undergo morphological and biochemical changes that lead to the formation of new cysts, which are eliminated in the feces, completing the cycle [53, 55, 56].

 The cytopathogenic effect caused by *E*. *histolytica* trophozoites is multifactorial. Intestinal flask-shaped ulcers, a hallmark of amoebic colitis, are characterized by severe damage to enteric cells as well as migration to the *lamina propria* and blood vessels [57, 58]. The contact between trophozoites and target cells appears to be the first step for cell lysis and phagocytosis. Several molecules are involved in this interaction, including the 260 and 220 kDa lectins and 112 kDa adhesin, which participates in the adherence to epithelial cells and erythrocytes [8, 59–63]. It has been proposed that for the initial amoeba contact or adhesion, surface carbohydrates on the target cell are recognized by specific molecules from the parasite. One of the better studied amoebic molecules is the Gal/GalNAc lectin, which mediates binding to host carbohydrate determinants that contain galactose and/or N-acetyl-D-galactosamine (GalNAc) [64, 65]. Adherence to colonic mucosa is conducive to the continued reproduction of parasites and tissue damage by the products secreted by amoebae, such as the pore-forming peptide amoebapore [66], which permits a massive influx of extracellular Ca^+2^ that is combined with the release of amoebic proteases at the site of contact, with the subsequent degradation of substrates. Once the targets are partially digested, the amoeba internalizes the cell debris and substrate fragments by phagocytosis [67]. Other proteins also contribute to host cell binding on target cells and destruction, such as phospholipases [68, 69].

### 2.1. Proteases of *E. histolytica* and Their Role in Virulence

Studies of *E. histolytica* proteinases (proteases) have mainly been performed in the strain HM-1:IMSS from axenically grown trophozoites. De la Torre et al. [70] isolated this strain from cysts of a Mexican patient suffering from intestinal amoebiasis. Most of the cellular and molecular studies of *E. histolytica* throughout the world, including the genomic sequence, have been performed with this strain. It has been cultured for years and passed through the liver of Syrian golden hamsters, an experimental model in which hepatic abscesses are reproduced to maintain and increase the virulence of *E. histolytica*. 

**Figure 1:**
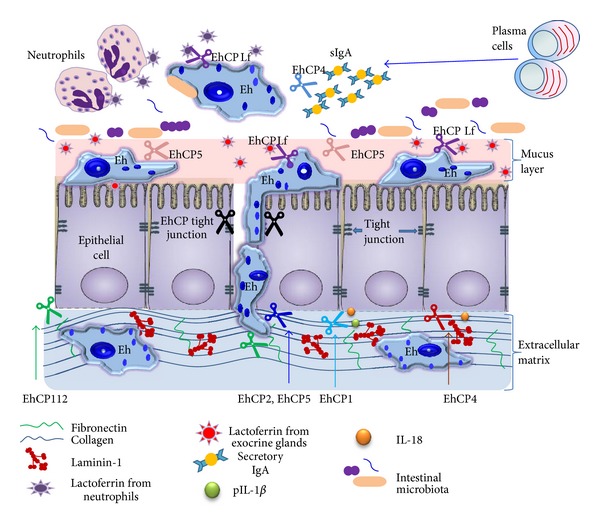
Proteases from *E. histolytica* as virulence factors during intestinal amoebiasis.

**Figure 2:**
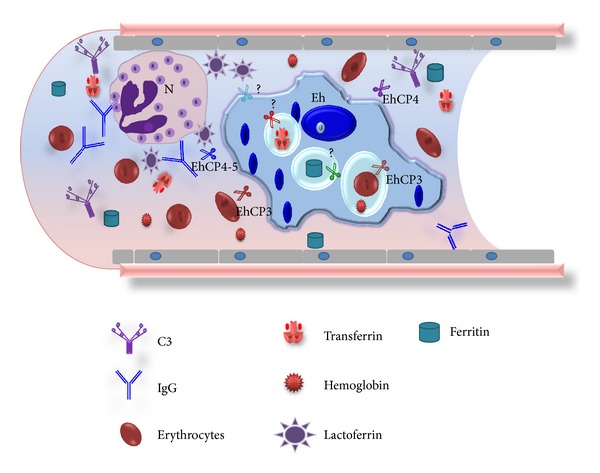
*E. histolytica *proteases participating during trophozoite transit in blood vessels.

**Figure 3:**
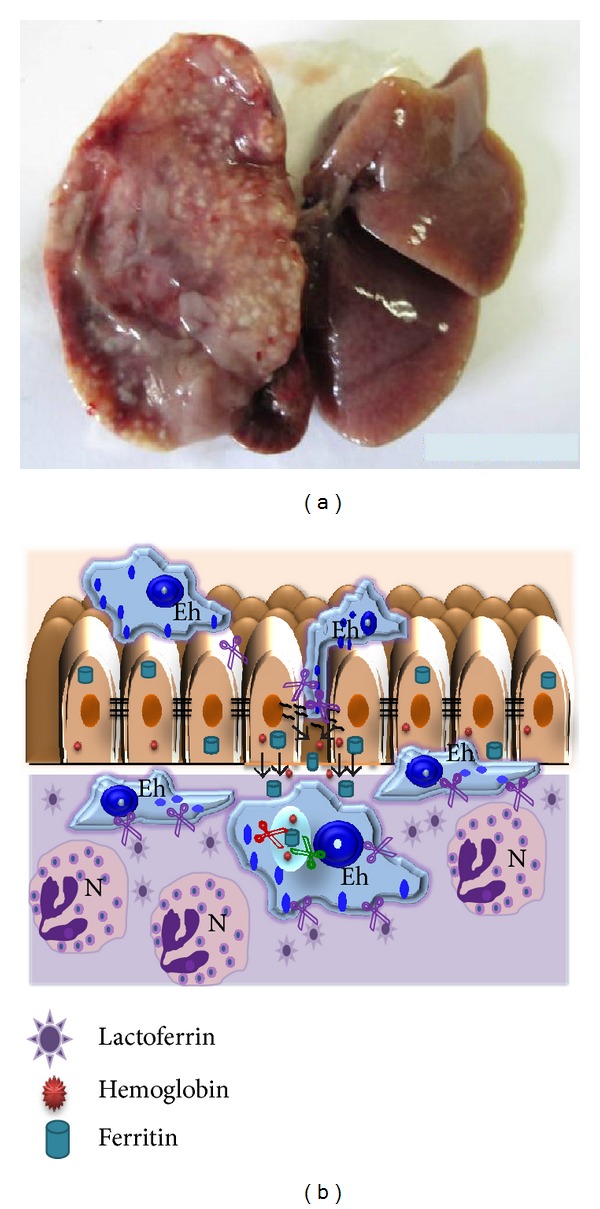
(a) Hamster amoebic liver abscess of eight days of infection (Photo kindly donated by G. Dominguez). (b) Proteases involved in the development of human amoebic liver abscess.

**Figure 4:**
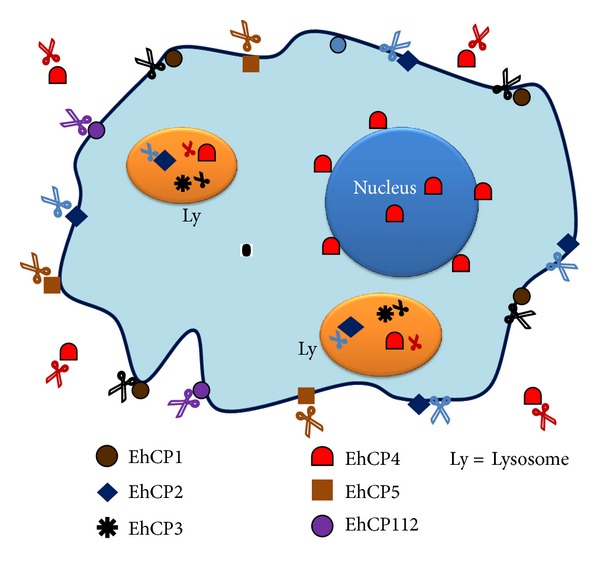
Localization of proteases in *E. histolytica*.

Cysteine proteases (CPs) are the predominant proteolytic activity associated with pathogenicity in *E. histolytica*, based on many studies in which the degradation of different substrates has been investigated, including purified proteins of the extracellular matrix (ECM), immunoglobulins, complement, and mucin. Figures 1 and 2 show the role of these proteases during intestinal amoebiasis and blood vessel transit of trophozoites, respectively. Animal model studies have confirmed that CPs have an important role in virulence; *E. histolytica* mutants impaired in genes encoding CPs have a diminished ability to produce hepatic abscesses [71, 72].  Figure 3 shows the role of proteases during amoebic liver abscess. EhCPs are expressed both intracellularly and extracellularly and are referred to as cathepsin-like enzymes because their structure is similar to that of cathepsin L; however, their substrate specificity resembles that of cathepsin B [34, 73–75]. Some proteases have been characterized as surface localized; hence, they have the potential to contribute to host tissue breakdown *in vivo *[74] (Figure 4). *E. histolytica* most studied proteases are summarized in Table 1.

#### 2.1.1. Purification and Cloning of the Main Cysteine Proteases from *E. histolytica*

Several genes encoding *E. histolytica*  CPs have been cloned. In 1990, Eakin et al. [76] amplified CP gene fragments from trophozoites by PCR from genomic DNA using degenerate oligonucleotide primers designed based upon amino acid sequences flanking the active site of CPs conserved in eukaryotic cells. The amplified DNA fragments were subcloned, and sequence analysis and alignment revealed significant sequence similarity to other members of the eukaryotic CP family (45% identical to chicken cathepsin L). The cysteine, histidine, and asparagine residues that form the catalytic triad are also conserved. Subsequently, [77] isolated three genes from the strain HM-1:IMSS, designated as *acp1*, *acp2,* and *acp3*. These genes have since been renamed, and the prefix “eh” has been added, so that these genes are now denoted *ehcp1* (*acp3*), *ehcp2* (*acp2*), and *ehcp3* (*acp1*). Next, we will review studies of the purification and cloning of *E. histolytica* CPs. Most of these studies were published before the establishment of an amoebic protease nomenclature or include experiments that only demonstrate degradation of the substrate; for these studies, we will focus on proteolytic activity and Mr. 

 By screening a genomic library from *E. histolytica*, [35] identified six genes encoding prepro-forms of CPs (*ehcp1*–*ehcp6*). The nucleotide sequences of these genes differed by 40%–85%, and three of the genes, *ehcp1*, *ehcp2*, and *ehcp5*, exhibited the highest levels of expression. Amoeba lysates were analyzed for CP activity against a synthetic substrate. Enzyme purification revealed that EhCP1, EhCP2, and EhCP5 are the main proteases, representing approximately 90% of CP expression. Tillack et al. [78] analyzed 79 of 86 genes encoding putative proteases by microarray hybridization. Of these, 50 encode CPs of various families, all of which belong to the clan CA. Interestingly, under standard culture conditions, the authors consistently observed the expression of only 20 genes. Very few differences were apparent among the isolates from asymptomatic and amoebic disease-affected individuals, even though the isolates had different geographic origins. Again, only three peptidase genes were expressed at high levels: *ehcp1*, *ehcp2,* and *ehcp5*. Accordingly, in the less pathogenic strain HK-9, the expression of EhCP5 was decreased by 2.3-fold. However, these *E. histolytica* isolates were grown under axenic conditions, and the expression profiles of CPs have been shown to adapt to different stimuli. This adaptation was confirmed in a transcriptional analysis of trophozoites isolated from the colons of infected mice versus trophozoites cultured *in vitro*. In contrast to what is observed in axenic cultured trophozoites, EhCP4, EhCP6, and EhCP1 were the most upregulated CPs during invasion and colonization of mice [79].

*EhCP1 (Amoebapain).*  A thiol-dependent protease was identified and partially purified by means of ammonium sulfate fractionation, gel filtration, and isoelectric focusing. The CP had Mr of 21 ± 2 kDa by gel chromatography. The maximal activity occurred at pH 4.4, but the CP was active at pHs of 3.4 and 8.5 [80]. Its proteolytic potential toward native substrates and bovine insulin B-chain was examined [81, 82]. The CP was purified by two gel chromatography steps, ion exchange chromatography on DEAE-cellulose, and affinity chromatography on organomercurial-Sepharose. The purified protease was monomeric with a Mr of 27 ± 2 kDa ; its activity required an arginine at the P2-position of the substrate. The protease was able to digest native collagen type-I, with an initial attack at the alpha 2-chain [6]. EhCP1 is located on the cell surface [34].

 By using an antiserum against the 27 kDa protease, a cDNA clone was obtained. Northern blot analyses suggested that the CP is more expressed in pathogenic isolates than in nonpathogenic isolates [83]. Accordingly, using a purification method similar to that described earlier, cathepsin B activity was obtained from virulent strains (HM-1:IMSS and Rahman), which yielded significantly more activity per milligram protein than less virulent strains (HK-9, Laredo, and Huff). These results suggest a correlation between amoebic cathepsin B and the pathogenesis of amoebiasis [26]. 

*EhCP2 (Histolysain).*  A one-step method for the purification to homogeneity of the histolysain of a soluble parasite extract by affinity chromatography to a synthetic substrate has been reported. The enzyme showed an apparent Mr of 26 kDa by SDS-PAGE and 29 kDa by gel chromatography. Its optimum pH varied widely with synthetic substrates, from 5.5 to 9.5. EhCP2 did not degrade type-I collagen or elastin but was active against cartilage proteoglycan and kidney glomerular basement-membrane collagen. EhCP2 also detaches cells from their substrates *in vitro* and could play a role in tissue invasion. This EhCP is located on the cell surface as well as in internal membranes [7].

*EhCP3 (ACP1).* EhCP3 (ACP1) was obtained by PCR amplification of *E. histolytica* genes using primers based on conserved structural motifs of eukaryotic CPs. It was initially reported to be present only in *E. histolytica *[77], but a related homologue gene (*edcp3*) with 95% identity has been detected in *E. dispar* [35, 84]. EhCP3 (308 amino acids) is synthesized as a preproenzyme, in which 13 amino acids form a signal peptide and 79 form a profragment (http://www.uniprot.org/uniprot/P36184). As a (His) 6-tagged protein, the mature enzyme migrates with an apparent Mr of 31 kDa [75]. The enzyme was cloned and expressed in baculovirus, and purified rEhCP3 was active in a broad pH range of 4.5–8.0 and had a neutral optimal pH. EhCP3 was sensitive to E-64, a specific CP inhibitor. The sequence of EhCP3 is most similar to that of cathepsin L, while its substrate preference for positively charged amino acids in the P2 position is consistent with cathepsin B [75]. Although EhCP3 is a relatively low-level transcript in HM-1:IMSS, after EhCP2, EhCP5, and EhCP1 [85], its expression is 100-fold higher in the Rahman strain [86].

 Using confocal microscopy with monoclonal antibodies (mAbs) to EhCP3, EhCP3 was localized primarily in the cytoplasm. Following erythrophagocytosis, EhCP3 colocalizes with phagocytic vesicles [75]. The presence of a homologous gene in *E. dispar*, in addition to its localization in *E. histolytica* phagosomes, has led to the hypothesis that the primary function of EhCP3 may be associated with the digestion of nutrients rather than virulence; however, further studies (i.e., knockdown experiments) are needed to clarify its function.

*EhCP4. *EhCP4 was described as a positive clone in a genomic library screened with an oligonucleotide probe derived from a sequence conserved between *ehcp1-3* and *ehcp5.* However, by Northern blot, the expression of *ehcp4 *and *ehcp6* was barely detected in amoebae grown *in vitro*. Analogous genes are present in *E. dispar*, but just as what occurs with *E. histolytica*, their expression is almost undetectable *in vitro* [35]. In a recent transcriptional analysis comparing *E. histolytica*  HM-1:IMSS isolated from mouse intestine with cultured amoebae from the same strain, both EhCP4 and EhCP6 were upregulated in trophozoites in the mouse gut (20–35 fold and 10-fold increased, resp.); in fact, *ehcp4* (*ehcp-a4*) was the most upregulated CP gene during invasion and colonization in a mouse cecal model of amoebiasis [79]. Upregulation of *ehcp4 in vivo* correlated with the finding that coculture of amoebae with mucin-producing T84 cells increased *ehcp4 *expression up to 6-fold. EhCP4 was cloned, including the prodomain and catalytic domain, for expression in bacteria. EhCP4 was purified and underwent autocatalytic activation at acidic pH but had greatest proteolytic activity at neutral pH. The calculated Mr of the mature enzyme was 23 kDa, but the apparent Mr was 26 kDa, as assessed by SDS-PAGE [14].

 Sequence alignment and computer modeling confirmed that EhCP4 is a member of Clan CA, C1A subfamily with a cathepsin L-like structure [73, 75, 87], similar to previously characterized EhCPs. However, unlike previously characterized EhCPs, which have a preference for cathepsin B or L substrates with arginine in the P2 position, EhCP4 displays a unique preference for valine and isoleucine at P2 [14]. EhCP4 is localized in the peri- and intranuclear regions as well as in variable-sized vesicles in the cytoplasm. In axenic amoebae, EhCP4 is primarily cytoplasmic, but in those isolated from infected mouse ceca, EhCP4 is found in both the nuclear and cytoplasmic extracts, suggesting a role for EhCP4 in the cell cycle or differentiation, as is the case for higher eukaryotic CPs with a nuclear localization [88–90]. Following coculture with colonic cells, EhCP4 appeared in acidic vesicles and was released extracellularly. Indeed, in the infected murine cecum, immunofluorescence staining of EhCP4 yielded strong signals in vesicle-like structures located in discrete surface areas. Thus, EhCP4 is apparently secreted *in vivo* [14]. 

 EhCP4 cleaves several host proteins *in vitro*, although in agreement with the different substrate specificity, the predicted digestion patterns differ from those of EhCP1. EhCP4 degrades C3, unlike EhCP1-mediated C3 processing, which produces an active C3b molecule [17, 18]. EhCP4 also degrades IgA, IgG, pro-IL-18, villin-1, and laminin-1 [14]. Thus, like EhCP1, EhCP4 could play an important role in destroying the integrity of tissues and evading the immune system and may contribute to the inflammatory response in amoebic lesions [91]. Based on the substrate preference and homology modeling, a specific vinyl sulfone inhibitor WRR605 was synthesized, which selectively inhibited rEhCP4. Treatment with WRR605 notably decreased the amoeba burden and intensity of cecal inflammation in a mouse cecal model of amoebiasis [14]. Together, these results suggest that EhCP4 is an important virulent factor in *E. histolytica* and a promising therapy target. 

*EhCP5.* A soluble protein of Mr 30 kDa with an affinity for membranes was purified from amoebic extracts. N-terminal sequencing and subsequent molecular cloning revealed that it was a member of the CP family of *E. histolytica*. The enzyme strongly associates with the membrane, retaining its proteolytic activity, and produces cytopathic effects on cultured monolayers. The 3D structure of EhCP5 revealed the presence of a hydrophobic patch that may account for membrane association potential of the protein. Immunocytochemical localization of the enzyme at the amoeba surface suggests a potential role in tissue destruction. The gene encoding EhCP5 is not expressed in the closely related but nonpathogenic species *E. dispar* [87, 92]. Trophozoites of the virulent strain transfected with an antisense gene encoding EhCP5 had only 10% activity but retained their cytopathic effect on mammalian cell monolayers and were incapable of inducing the formation of liver lesions in hamsters. However, this activity cannot be attributed only to EhCP5 because EhCP5 antisense mRNA also inhibited the expression of other CPs, which may be due to a high degree of sequence homology and conservation of residues critical for protease function [71, 93]. 

 EhCP5 has been confirmed to degrade a broad spectrum of biological and synthetic substrates such as mucin, fibrinogen, collagen, hemoglobin, bovine serum albumin, gelatin, IgG, Z-Arg-Arg-pNA, and Z-Ala-Arg-Arg-pNA but not Z-Phe-Arg-pNA. The identification of rEhCP5 as a CP was determined by using specific inhibitors [9]. EhCP5 activity and mRNA levels were analyzed in *E. histolytica* samples isolated from patients presenting different clinical profiles. The degree of virulence of the isolates, determined in hamster livers, correlated well with the clinical form of the patients and with the culture conditions. EhCP5 mRNA levels were also determined in fresh samples of amoebic liver abscesses. Differences were not observed in the levels of EhCP5 mRNA and CP specific activity among the cultured samples. However, different levels of EhCP5 mRNA were observed in amoebae freshly isolated from hepatic lesions. These results emphasize the importance of EhCP5 for amoeba virulence and the need for additional studies to validate the mechanisms involved in the pathogenesis of amoebiasis [94].

 EhCP5 was expressed in the bacterium *Escherichia coli* as a proenzyme and purified to homogeneity under denaturing conditions in the presence of guanidine-HCl. EhCP5 was renatured in buffer containing reduced and oxidized thiols, which led to a soluble but enzymatically inactive proenzyme. Further processing and activation was achieved in the presence of DTT and SDS. The recombinant enzyme (rEhCP5) was indistinguishable from native EhCP5 purified from amoebic lysates. Under reducing and nonreducing conditions, rEhCP5 exhibited Mr of 27 and 29 kDa, respectively, with the same optimal pH and similar specific activity against azocasein [9]. 

*EhCP112.*  EhCP112 is a papain-like proteinase [95]. It was first identified by using a mAb recognizing a protein present in wild-type trophozoites but missing in an adherence-deficient mutant. The mAb inhibited adhesion of amoebae to target cells, and a 112 kDa protein was identified by Western blot [59]. The DNA encoding this molecule was isolated from a genomic library, revealing that the 112 kDa adhesin is formed by two polypeptides (49 kDa and 75 kDa) encoded by two different genes separated by 188 bp. The 49 kDa polypeptide is a CP (EhCP112), whereas the 75 kDa protein has a domain involved in the adherence of amoeba to target cells (EhADH112); both proteins form a complex called EhCPADH. Within this complex, the peptides could be joined by covalent or strong electrostatic forces, and their proximity in the genome suggests a coregulated expression [60]. 

 EhCP112 (446 amino acids) is synthesized as a preproenzyme in which 19 amino acids form the signal peptide and 112 amino acids form the profragment. The mature protease has a putative Mr of approximately 34 kDa. EhCP112 contains a transmembrane segment domain characterized by the catalytic triad (C, H, and N), in addition to an RGD motif, which is an integrin attachment domain [95]. A previous report suggests that the majority of *E. histolytica* CP genes, including the *ehcp112* gene, are not expressed during *in vitro* culture [73]. However, Northern blot and RT-PCR assays indicate that the *ehcp112* gene is expressed in trophozoites [60, 96]. Different culture conditions or different pathogenicities of trophozoites could be responsible for the distinct results.

 EhCPADH is located in cytoplasmic vesicles and the plasma membrane and is also secreted. EhCPADH is translocated from the plasma membrane to phagocytic vacuoles during phagocytosis [60]. This implies a function in nutrient acquisition. However, EhCPADH has been associated with amoebae virulence. Antibodies against this complex inhibit adherence, phagocytosis, and destruction of MDCK monolayers by live trophozoites and extracts. Antibodies also greatly reduce the ability of amoebae to produce liver abscesses in hamsters [59, 60, 97–99].

 To elucidate the role of EhCP112 in virulence, it was cloned, expressed in bacteria and purified. rEhCP112 degrades collagen type I and fibronectin and destroys cell monolayers (MDCK). rEhCP112 also binds to erythrocytes and digests human hemoglobin [8]. rEhCP112 is active in a broad pH range (3–10), with the highest activity at pH 7.0 for azocasein and pH 6.0 for hemoglobin. EhCP112 was enzymatically active from 5 to 60°C with a maximum activity for both substrates at 37°C [95]. Interestingly, the EhCP112 enzyme is immunogenic in patients with amoebiasis [8].

#### 2.1.2. Proteases Involved in Trophozoite Adhesion

Leishmanolysin (Gp63) is a metalloendopeptidase that is essential for the virulence of *Leishmania major*. Leishmanolysin degrades ECM proteins during tissue invasion and prevents complement-mediated lysis of promastigotes [100, 101]. Two leishmanolysin homologues are encoded in the *E. histolytica* genome, but only one copy of the gene is present in the closely related *E. dispar*. The *E. histolytica* specific family member EhMSP-1 is a functional metalloprotease (MP) that is localized to the cell surface. Silencing EhMSP-1 expression dramatically increases amoebae adherence to live and dead cells, reducing mobility in cell monolayers and increasing phagocytosis. The knockdown of EhMSP-1 also reduces cell monolayer destruction but has no effect on the lysis of Jurkat lymphocytes. Thus, one possibility is that this phenotype could be the consequence of defective adherence because increased adherence may reduce the ability to move, which, in turn, causes the amoebae to be less able to destroy cell monolayers. The ultimate mechanism by which EhMSP-1 affects adherence remains unknown. However, it is known that EhMSP-1 knockdown does not affect steady-state surface abundance of previously identified adhesins such as the GalNAc-specific lectin and the serine-rich *E. histolytica* protein (SREHP) [102–104]. The ability of the CP inhibitor E-64 to almost completely block cell monolayer destruction suggests that EhMSP-1 silencing affects monolayer destruction indirectly and that EhMSP-1 itself likely does not directly degrade cell monolayers [91, 102, 105]. Apparently, EhMSP-1 plays no role in *in vitro* resistance to complement, in contrast to leishmanolysin.

 The rhomboid-like proteins are a large family of seven-pass transmembrane serine proteases (SPs) that were first identified in *Drosophila melanogaster* and whose active site lies within the lipid bilayer, allowing them to cleave transmembrane proteins [106, 107] and, hence, trigger signaling events [108]. Substrates of rhomboid proteases are largely single-pass transmembrane proteins with transmembrane domain containing helix-breaking residues [109]. The *E. histolytica* genome encodes four rhomboid-like genes, of which only a single gene, EhROM1, contains the necessary catalytic residues for proteolytic activity [110]. In resting conditions, EhROM1 is localized at the trophozoite surface and in internal punctuate structures; upon erythrophagocytosis, it relocalizes in internal vesicles and, during surface receptor capping, in the base of the cap. The heavy subunit of the Gal/GalNAc lectin (Hgl) is a substrate of EhROM1 *in vitro* [110]. EhROM1 knockdown leads to defects in both adhesion and phagocytosis but not cap formation or complement resistance. 

**Figure 5:**
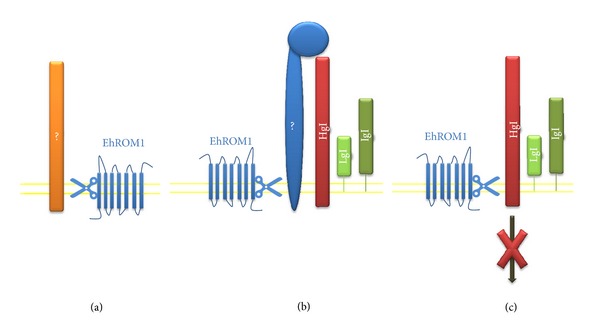
Proposed scenarios for how EhROM1 regulates parasite adhesion. EhROM1 may process an unknown adhesin (a) or a different substrate that masks the Gal/GalNAc lectin adhesion (b) or EhROM1 may play a role in signaling during the adhesion process by detaching the signaling integrin-like motif present in the cytoplasmic domain from the rest of the Gal/GalNAc lectin (c).

 Importantly, the reduced phagocytosis and adhesion phenotypes appear to be independent, implying that EhROM1 has distinct roles in both pathways. Interestingly, there were no significant changes in the expression or localization of the only known substrate, the heavy subunit of the Gal/GalNAc lectin, in EhROM1 knockdown parasites. Several scenarios have been proposed for how EhROM1 regulates parasite adhesion without affecting the expression or localization of the heavy subunit of the Gal/GalNAc lectin. EhROM1 may process a different adhesin or a different substrate that masks the Gal/GalNAc lectin adhesion, or it may play a role in signaling during the adhesion process by detaching the signaling integrin-like motif present in the cytoplasmic domain from the rest of the Gal/GalNAc lectin (Figure 5). Alternatively, EhROM1 may have a noncatalytic role in the adhesion process, as has been described for other rhomboid proteases [111, 112].

**Figure 6:**
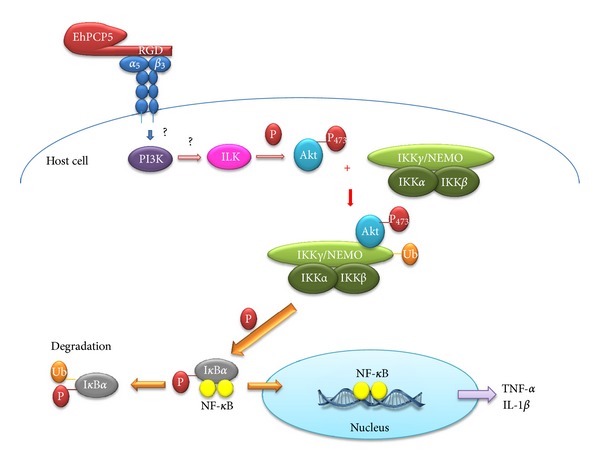
EhPCP5 stimulates NF*κ*B-mediated proinflammatory responses. Attached to *E. histolytica *surface or secreted, EhPCP5 binds to *α*(V)*β*(3) integrin through the RGD motif and triggers PI3K-mediated ILK activation. ILK phosphorylates Akt-473, which binds and induces the ubiquitination of NEMO. This activates the IKK*α*-IKK*β* complex that phosphorylate I*κ*B*α*. This phosphorylation event signals I*κ*B*α* ubiquitin-mediated degradation and, thereby, the release of NF-*κ*B into the nucleus, where it activates proinflammatory gene expression.

 Cell-adhesion processes and proteolytic mechanisms function in a coordinated manner to provide directed cell migration and are critical at the molecular level; adhesion receptors (integrins) are displayed on the cell surface and bind to ECM proteins, whereas proteases (e.g., MPs and CPs) modify or degrade ECM components. However, some proteases interfere with cell adhesion and/or migration in a nonproteolytic fashion [113–116]. In cell-adhesion proteins such as fibronectin, RGD motifs serve as ligand recognition sites for cell-surface receptors such as integrins. An RGD motif has been found in the pro-region of the lysosomal cathepsin X from higher eukaryotes, and apparently, this motif can mediate adhesion and migration processes via binding to integrins [114]. Only EhCP5, EhCP18, and EhCP112 present this motif [73]. The RGD motif is present in the pro-form of EhCP5 (EhPCP5); the RGD motif binds to *α*(V)*β*(3) integrin on Caco-2 colonic cells and stimulates NF*κ*B-mediated proinflammatory responses. Binding to this integrin triggers integrin-linked kinase- (ILK-) mediated phosphorylation of Akt-473, which binds and induces the ubiquitination of the NF-*κ*B essential modulator (NEMO) (Figure 6). As NEMO is required for the activation of the IKK*α*-IKK*β* complex and NF*κ*B signaling, these events markedly up-regulate proinflammatory mediator expression *in vitro* in Caco-2 cells and *in vivo* in colonic loop studies in wild-type and Muc2(−/−) mice lacking an intact protective mucus barrier. These results have revealed that the EhPCP5 RGD motif represents a novel mechanism by which trophozoites trigger an inflammatory response in the pathogenesis of intestinal amoebiasis [117]. 

 As mentioned earlier, EhCPADH is a surface complex that binds to target cells [59, 60, 99]. The role of EhCP112 in adhesion could be limited to its association with the adhesin EhADH112, although EhCP112 contains the RGD domain, suggesting that it binds to cellular integrins [8, 118]. The parasite may utilize surface-bound proteases to interact with integrins via the RGD motif to tether and propel along the endothelium, much like immune cells crawling along the vasculature to survey blood contents for pathogens [119].

#### 2.1.3. Proteases That Degrade Mucin

Mucous is the first layer that protects the colonic epithelium from potentially pathogenic viruses, bacteria, and parasites [120]. The ability of amoebae to invade may be inhibited by the binding of mucin to lectin, which inhibits lectin activity, and by the physical barrier between the parasite and the intestinal epithelium created by mucin [64]. MUC2 is the major gel-forming mucin secreted by goblet cells of the small and large intestines and is the main structural component of the mucus gel [121, 122]. To gain access to the underlying epithelial cells, amoebae must first breach the protective mucous layer. This phenomenon has been observed in biopsies from patients with acute amoebic colitis [58] and has been confirmed in an *ex vivo* human colonic model of amoebiasis; after just two hours of incubation, the mucus layer was no longer observable, suggesting that it had been removed by amoebae [123]. *E. histolytica*  secreted CPs are responsible for degrading MUC2 [124]; cleavage at the major cleavage site is predicted to depolymerize the MUC2 polymers, thereby disrupting the protective mucus gel [125]. In addition, the parasite encodes glycosidases that degrade mucin oligosaccharides [126]. *E. histolytica* may use the concerted actions of glycosidases and proteases to disassemble the mucin polymeric network. 

 Trophozoites with reduced CP activity are ineffective in degrading and crossing the protective mucus layer produced by cell lines in culture [31, 93]. However, controversy remains about the effector protease(s) responsible for mucin degradation. Bioactive recombinant EhCP5 is capable of degrading purified native mucin [9, 31]. However, an EhCP5/amoebapore-silenced strain was able to cross the mucus barrier in an *ex vivo* colonic model but was unable to migrate within the mucosa. This behavior suggests that other proteases produced by the EhCP5/amoebapore-silenced strain appear to be involved in the removal of the colon mucin gel [123]. Alternative effector proteases include EhCP1, EhCP2, and EhCP4 because their expression is increased after trophozoites are exposed to mucin [14, 127]. This suggests that the specific composition of mucin may affect the ability of *E. histolytica* CPs to degrade the mucous layer.

#### 2.1.4. Alterations in Microvilli and Tight Junctions

After the mucus, the tight junction (TJ) complex constitutes the first barrier against the paracellular penetration of intestinal microorganisms. This barrier is formed by the plasma membrane-spanning proteins claudin and occludin, which are associated with the cytoplasmic proteins ZO-1, ZO-2, and ZO-3. *E. histolytica *is able to disturb the TJ complex from human colonic adenocarcinoma T84 and MDCK T23 cells. Trophozoites decrease the transepithelial electrical resistance in T84 cells and, to a lesser extent, in MDCK cells [128]. The same group of researchers subsequently observed the dephosphorylation of ZO-2, loss of ZO-1 from ZO-2, and degradation of ZO-1 when amoebae were cocultured with T84 cells [129]. By contrast, [130] analyzed the interaction between trophozoites and human Caco-2 cell layers and identified a crucial role for amoebic CPs in the prevention of villin proteolysis and associated microvillar alterations through the treatment of Caco-2 cell layers with inhibitors that completely block CP activity. Moreover, trophozoites of strains pSA8 and SAW760, which have strongly reduced CP activity, exhibited reduced proteolysis of villin in coculture with enteric cells, indicating that villin proteolysis is an early event in the molecular cross-talk between enterocytes and amoebae that causes the disturbance of microvilli. Both EhCP1 and EhCP4 are capable of degrading human villin *in vitro*, and thus they could play an important role in destroying the integrity of microvilli *in vivo* (Che He 2010). In addition, [131] reported that TPCK and TLCK, two SP and CP inhibitors, can affect *E. histolytica* proteases and prevent proteolysis of ZO-1, ZO-2, and villin in Caco-2 cells. Taken together, these results indicate that amoebae use their proteinases to overcome microvilli and tight junction barriers during the invasion of enteric cell layers. 

#### 2.1.5. Proteases That Degrade the Extracellular Matrix

Cysteine proteases have been demonstrated to act on a variety of substrates *in vitro*.  In the host, parasite proteases facilitate tissue penetration by dissolving the intestinal mucosa and extracellular matrix (ECM) and freeing epithelial cells [7, 11, 27, 125, 130, 132–134]. More than 80% of patients suffering from amoebiasis express antibodies to trophozoite CPs [135]. Interestingly, purified enzymes that degrade one of the ECM proteins have been found to cleave other ECM proteins, generally fibronectin, collagen, and laminin. The interaction of amoebae with ECM components results in the proteolysis and destruction of the connective tissue. 

 For 30 years, proteases degrading native type-I and type-III collagen fibers have been studied in cultured *E. histolytica*, although CPs were purified more recently. Collagenase is more active against type-I collagen. Three major fragments of 75, 50, and 25 kDa were obtained from type-I collagen upon incubation with amoebae. After 3 h of incubation, smaller fragments of degraded collagen were observed, possibly due to the action of other proteolytic enzymes [136]. In other work, FPLC-anion-exchange and chromatofocusing chromatography were used to purify the major neutral proteinase from secretions of the cultured  *E. histolytica* HM-1:IMSS strain. This proteinase is a CP with a subunit Mr of approximately 56 kDa, a neutral optimum pH, and a pI of 6. The importance of this enzyme in extraintestinal amoebiasis is suggested by its ability to degrade a model of connective tissue ECM as well as purified fibronectin, laminin, and type-I collagen. The enzyme caused a loss of adhesion of mammalian cells in culture, most likely because of its ability to degrade anchoring proteins. Experiments with a peptide substrate and inhibitors indicated that the proteinase preferentially binds peptides with arginine at P-1. It is also a plasminogen activator and could thus potentiate host proteinase systems [11]. A 56 kDa band was identified by polyacrylamide-gelatin gel electrophoresis in 10 of 10 isolates from patients with colitis or amoebic liver abscesses but in only 1 of 10 isolates from asymptomatic patients. Pathogenic isolates appear capable of releasing significantly larger quantities of this CP, which is released during the course of invasive disease, as demonstrated by the presence of circulating antibodies detectable by enzyme-linked immunosorbent assay (ELISA). These studies support the importance of the 56 kDa CP in the pathogenesis of invasive amoebiasis [91]. 

 The collagenase activity was mainly observed in electron-dense granules. These granules were induced and secreted in response to the incubation of amoebae with collagen type I *in vitro*. A specific collagenase activity with Mr of 72 kDa was identified in crude extracts. We observed this activity in electron-dense granules; this activity could be related to actin cytoskeleton function because the cytoskeleton-altered amoeba strain BG-3 derived from the HM-1:IMSS strain exhibits lower collagenase activity [12, 137]. Incubation of amoebae with type-I collagen not only promotes collagenase activity but also increases the secretion of other CPs [15], and together with Ca^2+^, type-I collagen can induce the activation of several amoebic genes related to virulence factors, such as amoebapore C and EhCP5, along with the stress-induced protein HSP70 and the ribosomal protein L27a [138]. In addition, a correlation between collagenolytic activity and virulence was observed when the levels of activity of different strains (virulent HM-1:IMSS and the less virulent 200-NIH and HK-9) [139–141] or of other virulence factors [142] were compared, suggesting that collagenase may play a role in the pathogenesis of amoebiasis. In a recent study, Chavez-Munguia et al. [143] demonstrated that electron-dense granules contain multiple CP activities. 

 Recently, the behavior of wild-type and EhCP5-silenced *E. histolytica* was compared on a 3D-collagen matrix and within human colon fragments for fibrillar collagen cleavage and migration. Interstitial collagen fibers within the connective tissue of the human colon, visualized by multiphoton and second harmonic generation signal imaging, presented a dense scaffold at the subepithelial level and a loose meshwork within the chorion. To penetrate the tissue, wild-type *E. histolytica* migrated on the dense scaffold that remained intact, reached the crypt of Lieberkhün, migrated along, and then disorganized the loose scaffold to escape into the mucosa. However, *in vitro*, EhCP5 was not required for collagenase activity and migration through the matrix but was necessary within the tissue environment for collagen meshwork remodeling and subsequent invasion. The data indicate that subsequent steps of the invasion relay with ECM destruction require human components that are induced or activated in the presence of EhCP5 [10].

 The major 27–29 kDa CP has been studied since 1989. This cystatin-inhibitable CP degrades the ECM proteins collagen types IV and V as well as laminin and fibronectin with different velocities and specificities under native conditions. Whereas the degradation of fibronectin and laminin proceeds rapidly, collagen breakdown occurs slowly and incompletely. This CP represents, by far, the highest portion of soluble proteolytic activity in *E. histolytica*  and is sufficient to destroy the host ECM [13]. Similar results have been reported for the 27 kDa CP with respect to the binding specificity for immobilized laminin over collagen and fibronectin. Inactivation of the CP with the active-site inhibitor E-64 abolishes laminin binding by the enzyme, and conversely, laminin inhibits the cleavage of a fluorogenic dipeptide substrate of the CP, suggesting that the substrate binding pocket of the enzyme binds to laminin. Furthermore, the addition of laminin but not fibronectin or collagen to amoebae significantly reduces amoebic liver abscess formation in severe combined immunodeficient (SCID) mice, further supporting the assumption that CPs play an important role in amoebic pathogenesis [21]. 

 An *in vitro* model was developed to analyze the adhesion to and cleavage of human fibronectin-covered surfaces. This model revealed the specificity of the binding and occurrence of structural and biochemical events in amoebae that participate in and promote adhesion to the substrate and its degradation. Similar results were obtained with laminin and Matrigel [144, 145]. A putative amoebic fibronectin receptor with Mr of 37 kDa was identified. Adhesion to fibronectin triggers the release of proteases, which facilitates the local degradation of the substrate [144, 146]. Some of the proteases may generate fragments with chemotactic and chemokinetic properties that are able to promote the binding and locomotion of amoebae [147]. In later studies, ECM proteins have been shown to be cleaved *in vitro* by identified CPs; collagen is degraded by EhCP1 [6], EhCP2 [7], and EhCP112 [8], fibronectin by EhCP112 [8], and laminin by EhCP4 [14].

#### 2.1.6. Cytopathic Activity

Several studies have shown a direct correlation between *E. histolytica* CPs and tissue damage; as CP activity decreases, tissue damage decreases. This relationship has been tested using trophozoites with low levels of CPs in an amoebic liver abscess hamster model [71] or in human colonic xenografts [22]. The inhibitor of CP activity E-64 reduces abscess formation in SCID mice [148], and specific inhibitors of EhCP4 reduce cecal inflammation [14], or specific inhibitors of EhCP1 in human colonic xenografts [17]. In the opposite strategy, overexpression of *ehcp5* but not ehcp1 or *ehcp2* significantly increased abscess formation in gerbils [149].

 The results of *in vivo* experiments reflect all of the pleiotropic effects of EhCPs in the pathogenic mechanism of amoebiasis. Some research has sought to probe the direct cytopathic effect of each EhCP, but the resulting data are controversial. Cytopathic activity, as measured by *in vitro* monolayer disruption, was increased in EhCP5- and EhCP2-overexpressing *E. histolytica* [149, 150]. However, silencing of the major *ehcp* genes (*ehcp1, ehcp-2, ehcp-5,* and *ehcp7*) or a reduction of expression to only 10% of the regular levels of CP activity did not influence trophozoite-induced cell monolayer disruption, suggesting that these peptidases are dispensable for cytopathic activity [93, 151]. All of these results were obtained using live intact trophozoites; however, lysates of virulent *E. histolytica* have also been shown to destroy monolayers [152]. Lysates of amoeba with only 10% of the total EhCP activity exhibited very poor activity against monolayers. This result was confirmed using the general CP inhibitor E-64; while CPs of viable trophozoites are responsible for 50% of cytopathic activity, this inhibitor completely blocks the cytopathic activity of *E. histolytica* lysates [150]. The effects of E-64 suggest that CPs are not the main virulence factor involved in the destruction of monolayers by intact trophozoites, but they must play some role, particularly whenever there is also some lysis of the trophozoites [93]. 

 Nevertheless, these data should be interpreted cautiously because monolayer destruction was assayed; these assays cannot distinguish between monolayer releases reflecting ECM degradation from actual cell death [57]. The investigation of the response of cultures of a human liver sinusoidal endothelial cells (LSECs) line to incubation with virulent or virulence-attenuated *E. histolytica *shed some light on this controversy. The data obtained suggest that amoeba interference of the integrin-focal adhesion signaling pathway plays a role in the induction of human cell retraction and death. Using silenced strains, the authors reported that this phenomenon occurred independently of the cytolytic amoebapore but required galactose-inhibitable parasite adhesion, likely involving amoebic Gal/GalNAc lectin, and active *E. histolytica* CPs [153]. This is in agreement with the finding that secretory CPs of *E. histolytica *are capable of inducing anoikis in MDA-MB-231 cells. Anoikis is a specialized type of apoptosis that mammalian epithelial, endothelial, and various other cell types experience upon their detachment from the extracellular matrix (ECM). Based on the results of these two studies, a model of pathogenesis of amoebiasis may be proposed involving CP-mediated degradation of ECM-cell and cell-cell attachments, thereby inducing anoikis of hepatocytes and enterocytes, which could lead to liver abscess and colonic ulcer formation. However, further investigations are needed to elucidate the role of anoikis in amoebiasis [154].

#### 2.1.7. Proteins of the Immune System Are Cleaved by Cysteine Proteases

*Immunoglobulins.* Secretory IgA (sIgA) Abs are considered a first line of specific defense against natural infections in the vast area occupied by mucosal surfaces [155]. sIgA functions *in vivo* by reducing mucosal colonization by pathogens and neutralizing diverse toxins and enzymes [156]. *E. histolytica* elicits a local immune response, in which an increase in specific IgA is detectable in several compartments associated with the mucosa [157, 158]. IgA inhibits the *in vitro* adherence of amoebae to epithelial cell monolayers by recognizing several membrane antigens [157, 159], reduces proteolytic activity [160], and has amoebicidal action [161]. Several lines of evidence indicate that sIgA protects against *E. histolytica* infection [157]. Children with stool IgA specific for lectin appear to be protected from intestinal infection [162], and Gal-lectin heavy subunit-specific intestinal IgA is sufficient to provide immunity against *E. histolytica* intestinal infection in a baboon model [163].

 Interestingly, several lines of *in vitro* evidence support that *E. histolytica* strikes back by using its surface and secreted CPs to degrade host sIgA. When serum and sIgA are exposed to viable axenic trophozoites (strain HM-1:IMSS), a parasite sonicate, or conditioned medium by incubation with live amoebae, sIgA is completely degraded, and proteinase activity is maximal at a neutral pH and is completely inhibited by E-64 [16]. Serum and sIgA are susceptible to degradation by amoeba surface-associated CPs; both sIgA1 and sIgA2 are degraded in a similar fashion by surface amoeba proteases. However, sIgA2 is functionally more resistant to proteolysis than sIgA1 [156]. The CP identity was not determined, but it could be a 70 kDa protease [16]. EhCP4 was recently shown to degrade IgA *in vitro* [14]; however, it is unclear if it is the same protease or if *E. histolytica* has more than one IgA-degrading protease. In fact, Garcia-Nieto identified this activity as a surface protease, and EhCP5, unlike EhCP4, is found in the amoeba plasma membrane [87], leading the authors to suggest that this enzyme could be the main protease involved in sIgA degradation [156]. 

 Specific antiamoeba IgG responses are developed in >95% of patients with amoebiasis or even in individuals with *E. histolytica* asymptomatic colonization [164]. However, it is difficult to ascribe a protective role to IgG because the level of Ab response correlates with the length of disease not with the clinical response to infection [164], and mixed results have been obtained with animal models [165, 166]. This may be because IgG is cleaved by amoebic CPs [34]. A 56 kDa proteinase purified from *E. histolytica* cleaved polyclonal human and monoclonal murine IgG in a dose-dependent manner. Intact trophozoites also cleave IgG. The resulting cleaved mAb bound to trophozoites with lower affinity than the uncleaved antibody, indicating a decrease in affinity and limiting the effectiveness of the host humoral response to the parasite [11, 167]. The identity of the CP in this study is also unknown, although later *in vitro *studies with purified recombinant enzymes have demonstrated that EhCP1 [17], EhCP4 [14] and EhCP5 [9] degrade IgG.

 Immunoglobulin cleavage in amoebiasis could decrease the affinity of the antibody for antigen, or if the Fc portion was removed, trophozoites could evade the immune system by coating their surface molecules in Fab fragments [133]. This would prevent the activation of complement by the classical pathway and attack from immune cells bearing corresponding Fc receptors [34, 119].

*Complement.* Complement (C) is activated when zymogens in serum are cleaved and produces an enzyme cascade that results in nonspecific binding of C components to pathogen surfaces. The two primary functions of C are to directly lyse foreign cells by the membrane attack complex C5b-9 and to opsonize pathogens with C molecules [119]. C3 is the central molecule of the alternative pathway; hydrolysis of C3 generates its active fragments C3a and C3b. Covalent deposition of C3b on nearby surfaces triggers a cascade of events that end in membrane attack complex-mediated lysis [168].

 The interaction of amoebae with C seems to be quite complex. In a comparative *in vitro* analysis of several amoebic strains, a tight correlation between C resistance and the degree of amoebic virulence was observed [169]. However, during liver abscess development in hypocomplementemic animals, tissue damage and parasite survival increase [170]. *E. histolytica* can resist lysis mediated by the C system through extracellular C activation through the action of EhCPs. The 56 kDa secreted neutral CP activates the alternative pathway of C by cleaving C3 in the fluid phase. CP action is similar to C-derived C3 convertases, and no further degradation occurs. Therefore, the C3b-like molecule produced is hemolytically active, as demonstrated by its ability to accelerate activation of the alternative pathway of C in rabbit erythrocytes [18]. Only C-sensitive nonpathogenic *E. histolytica* are lysed by this fluid phase-activated C3b-like molecule, while pathogenic strains are resistant [171]. Pathogenic amoebae most likely achieve this by inhibition of surface deposition of the membrane attack complex [119, 172]. The protease involved has not been identified; however, EhCP1 has been shown to cleave C3 in a manner identical to that of the secreted 56 kDa native proteinase [18] to generate the *α*′ subunit and possibly form an active C3b molecule [17]. Likewise, EhCP4 cleaves C3 and produces a fragment with a size similar to that of C3b; however, unlike EhCP1, EhCP4 degrades C3b-like ones [14]. Further studies are needed to determine whether C3 is actually activated *in vivo* by EhCP1 or by an as-yet unidentified protease or if it is actually degraded by EhCP4.

C3a and C5a, the small cleavage fragments released by C activation, are potent mediators of inflammation and anaphylatoxins [173]. The same extracellular proteinase (56 kDa) of pathogenic *E. histolytica* is capable of limiting a potential host defense mechanism by degrading C3a and C5a [19]. The anaphylatoxin blockade decreases immune detection in the blood and reduces inflammation in amoebic lesions, partially explaining the lack of severe inflammation in advanced liver and intestinal lesions [119].

*Molecules Involved in Inflammation.* In a SCID mouse-human intestinal xenograft model, infection with *E. histolytica* trophozoites elicits a robust inflammatory response from the grafted tissue, characterized by strong IL-1*β* and IL-8 expression, an early neutrophil influx, and extensive damage to the intestinal graft [174]. Trophozoites with reduced CP activity fail to induce intestinal epithelial cell production of the inflammatory cytokines IL-1*β* and IL-8 and cause significantly less gut inflammation and damage to the intestinal permeability barrier [22]. Purified amoebic CPs possess IL-1*β* converting enzyme (ICE or caspase-1) activity *in vitro*, cleaving recombinant human pIL-1*β* into a biologically active form of IL-1*β* [22]. These purified proteases are most likely EhCP1 and EhCP2 [21, 22]. In this work, the authors postulate that amoebae first bind to intestinal epithelial cells and then lyse those cells through the action of amoebapore [175, 176]. The lysed cells may release pIL-1*β*, which could then be activated by extracellular amoebic CPs with ICE activity and further amplify the inflammatory process in amoebic colitis [22].

IL-18 is expressed in intestinal epithelial cells [177] and is a coinducer of the Th1 response. The resulting stimulation of IFN*γ* then activates macrophages, the major cell capable of killing *E. histolytica* trophozoites [178]. IL-18 and IL-1*β* maturation requires cleavage by caspase-1. However, in contrast to the activation of proIL-1*β* by amoebic lysates, purified rEhCP5, rEhCP1, and rEhCP4 cleave proIL-18 and mature IL-18 to biologically inactive fragments [14, 17, 20]. These contradictory findings, in which EhCPs have opposite effects in two proinflammatory cytokines, have not been explained; the balance between these EhCPs is likely controlled by a complex interplay of parasite and host molecules.

#### 2.1.8. Proteins Containing Iron Are Degraded by Amoebic Proteases for Use as Iron Sources for Growth

Iron is a vital element for the survival of almost all organisms. However, under physiological conditions, Fe^3+^ is not soluble, and Fe^2+^ is soluble but toxic and readily oxidizes to Fe^3+^. To increase solubility, avoid toxicity, and keep iron away from intruders, this element is normally complexed to proteins; thus, the free iron concentration is far too low to sustain the growth of intruders. However, successful pathogens are able to scavenge iron from host proteins [179–183]. *E. histolytica*, as well as other amitochondriate protists (e.g., *Tritrichomonas, Trichomonas*, and *Giardia*), requires particularly high amounts of extracellular iron *in vitro* (~100 *μ*M), surpassing that of the majority of both eukaryotic and prokaryotic cells (0.4–4 *μ*M) [184]. This high iron requirement is attributable to the heavy reliance of their energy metabolism on Fe-S proteins [185–187].

**Figure 7:**
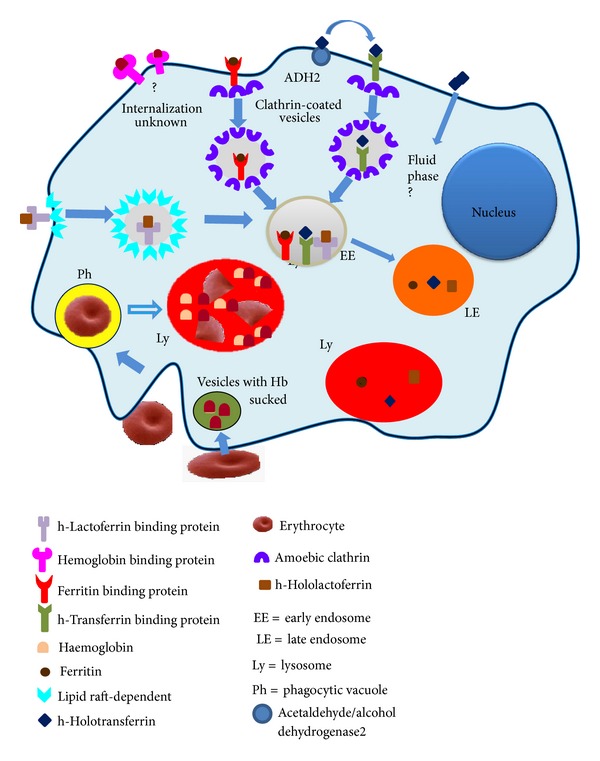
Host proteins containing iron are internalized and degraded by amoebic proteases for use as iron sources for growth.

We have reported that *E. histolytica* trophozoites are able to use four of the human iron-containing proteins as iron sources for the parasite's growth in axenic culture medium in which ferric ammonium citrate was substituted by the ferrous- or ferric-protein under investigation. These proteins are hemoglobin, transferrin, lactoferrin, and ferritin [185]. In all cases, amoebae were able to endocytose and cleave the protein to obtain the needed iron (Figure 7). The use of these proteases by trophozoites could be considered a virulence factor because the pathogens seek out host iron to survive in the hostile host environment [179, 182, 183, 188, 189]. 

*Hemoglobin.* Hemoglobin (Hb) is a globular protein that is present at high concentrations in erythrocytes or red blood cells (RBCs). The function of Hb is to trap oxygen in the lungs and transport it through the blood to tissues and cells. In adult mammals, Hb is composed of two alpha and two beta chains, each containing one heme prosthetic group; therefore, there are four Fe^2+^ atoms in the Hb molecule, which has Mr of 64.5 kDa [190]. Hb uptake by *E. histolytica *trophozoites occurs by disrupting the RBC cytoplasmic membrane with surface hemolysins and phospholipases. The major amoebic hemolytic activity has been characterized in rat RBCs; this activity was detected in a vesicular fraction [191, 192]. An alkaline phospholipase has also been associated with virulence [193]. 

In *E. histolytica*,  there is little information regarding how iron is obtained from Hb. This parasitic protist is extremely active as a phagocytic cell; once phagocytosed, human RBCs are broken down by amoebae. Chévez et al. [194] described complete RBC digestion in ~6–8 h by Perl's staining experiments. Quantitative digestion assays using diaminobenzidine staining to visualize RBCs revealed that, after 9 h of RBC phagocytosis, Hb was thoroughly degraded [194–196]. Several researchers have studied the role of amoebic hemoglobinases in the cleavage of different types of Hb. The degradation of native bovine Hb at pH 7.6 by extracted proteinases from different monoxenic strains was observed [197]. Thirty-five years ago, two proteinases against native bovine Hb were purified [23]: one of 41 kDa, with optimal activity at pH 3.5, and another of 27 kDa, with optimal activity at pH 6.0. Subsequently, a cytotoxin of 22 kDa with strong proteolytic activity against denatured Hb at an optimal pH of 4.5 was described, and a cathepsin B of 16 kDa that was active against native and denatured Hb was purified [24–26]. Perez-Montfort et al. [24] identified two proteins of 32 and 40 kDa that were able to degrade denatured Hb.

 Our group has described three proteases of 21, 82, and 116 kDa in extracts of *E. histolytica *HM-1:IMSS. These proteases were able to degrade human, bovine, and porcine Hbs, mainly at pH 7.0, and were inhibited by PHMB, E-64, NEM, and IA, all of which are specific CP inhibitors [27]. Becker et al. [198] reported a 30 kDa protease in vacuoles that previously contained phagocytosed RBCs; electrophoretic analysis revealed the incorporation of Hb monomers into trophozoites. In parallel to the decrease in human Hb during RBC digestion, X-ray analysis revealed a loss of iron content [198, 199]. *In vitro* assays have demonstrated that purified recombinant EhCP112 and EhCP5 are able to degrade Hb [8, 9].

*Transferrin.* In mammals, iron is mainly transported by transferrin (Tf), a protein found in serum and lymph that delivers iron to all sites, mainly to tissues with active cell division and bone marrow erythroid cells synthesizing Hb. Tf is part of a family that also contains lactoferrin. Tf has a *K*_*d*_ for Fe^3+^ of 10^−22^ M and is extremely stable against degradation when saturated. Tf is a glycoprotein of 80 kDa with two lobes, each containing one binding site with differing affinity for Fe^3+^. TfR1 is a dimeric glycoprotein of approximately 90 kDa per subunit that is expressed in nearly all cells [200, 201].

 Interestingly, one of the amoebic receptors for holoTf is the acetaldehyde/alcohol dehydrogenase-2, an enzyme that requires iron. HoloTf is endocytosed through clathrin-coated vesicles and transported to lysosomes, likely losing the first iron in early endosomes and the second in lysosomes due to the acidic pH [28, 202]. To determine whether trophozoites possess cytoplasmic or secreted proteases that can degrade holoTf, total extracts and culture supernatants (SN) of medium with ferric citrate or in the absence of iron were analyzed for holoTf cleavage. Four bands of holoTf degradation corresponding to 130, 43, 20, and 6 kDa were observed in the extracts. In contrast, five bands of 130, 70, 50, 35, and 30 kDa were observed in the SN. All of the proteolytic activities were of the cysteine type. Secreted CPs could play a key role in cleaving Tf when amoebae travel by the portal vein to the liver and when, upon remaining in the liver, produce hepatic abscesses [28].

*Lactoferrin.* Lactoferrin (Lf) is a glycoprotein from the innate immune system that is secreted to mucosae; it is abundant in colostrum and milk. Lf is secreted without iron (apoLf) by the secondary granules of neutrophils at the infection site; thus, it is a marker of inflammatory bowel diseases (IBDs) [203]. One of the functions of Lf is to chelate iron to make it unavailable to intruders. Lf is a single polypeptide chain that is folded into two lobes; like Tf, each lobe can bind one Fe^3+^. The degree of Lf glycosylation determines its resistance to proteases and to very acidic conditions. Apo-Lf has a higher avidity for iron than apo-Tf. HoloLf releases iron only in very acidic environments (e.g., pH < 4), and its conformation changes according to the saturation state. When saturated, Lf is more stable and resistant to proteolysis [204–206]. 

 HoloLf can be used as a sole iron source for *in vitro* growth by *E. histolytica* trophozoites in a similar fashion to that observed for ferric citrate. HoloLf was recognized by two proteins (45 and 90 kDa) located in the amoebic membrane, and its binding was specific [29]. HoloLf enters the amoeba by a clathrin-independent via (possibly caveolae-like structures). Following endocytosis, holoLf is found in vesicles similar to early endosomes and is then delivered to late endosomes and lysosomes. Delivery of holoLf to lysosomes may be required for its digestion by proteases and iron release, which only occurs in a very acidic milieu. CPs of 250, 100, 40, and 22 kDa from amoebic extracts cleaved holoLf at pH 7; however, the activity increased considerably at pH 4 [29]. In acidic lysosomes, the iron from holoLf is likely released, and the protein is degraded by CPs. Culture SN did not contain proteolytic activity against holoLf. Whether *E. histolytica* contains a reductase capable of changing the iron oxidation state remains unknown. This mechanism seems to be shared by other parasites, such as *Tritrichomonas foetus *[207]. As amoebae develop in the intestinal mucosa where Lf is found, this protein could be the iron source for the parasite at the beginning of infection, in addition to iron-containing bacterial proteins.

*Ferritin.* Due to the toxicity of iron, all life forms must have a mechanism to store/scavenge excess iron. Human ferritin is a major cytosolic protein with the capacity to capture up to 4,500 iron atoms. When the intracellular iron level increases, ferritin sequesters iron inside its cavity to detoxify the cell and prevent damage. Ferritin is abundant in the liver, which stores ~50% of the body's total iron reserves. The mammalian ferritin family generally consists in spherical proteins. Each 474 kDa molecule consists of 24 subunits that are either heavy (H) or light (L) with a molecular mass of ~21 and ~19 kDa, respectively [208–211].

 Ferritin uptake by amoeba may be mediated by a binding protein because it is concentration and time dependent, highly specific, and saturable at 46 nM ferritin. *E. histolytica* can cleave ferritin into several fragments. Three neutral CPs (100, 75, and 50 kDa) were observed to degrade ferritin in culture extracts. Ferritin entrance is constrained by inhibitors of clathrin-coated pits, and after 30 min of incubation, ferritin colocalized with an anti-rat LAMP-2 Ab in lysosomes [30]. The liver invasion by *E. histolytica *is poorly understood. Once liver cells are destroyed by amoebic enzymes, ferritin can be released and may be endocytosed by trophozoites and used as a source of iron and nutrients to form hepatic abscesses. In the liver, amoebae may also use Hb as an iron source; however, ferritin can provide up to 1,000-fold more iron than Hb. The capacity of *E. histolytica* to utilize ferritin as iron source may well explain its high pathogenic potential in the liver.

## 3. *Entamoeba histolytica* Related Strains

Virulence is a complex phenomenon that depends on two general properties: the invasiveness, or ability of microorganisms to multiply and to cause localized tissue destruction, and toxigenicity, or the ability to produce and secrete substances that can cause distant lesions. However, the virulence of *E. histolytica* related strains likely depends mainly on the tissue-damaging potential of individual trophozoites and the number of invasive amoebae in the infected host [212]. 

 The role of amoebic proteases as responsible for tissue destruction in amoebiasis has been discussed in the previous section [7, 13, 91]. Amoebic proteases have been shown to degrade several tissue components such as collagen, fibronectin, and laminin [13, 91]. The pathogenicity and degree of virulence of various amoebic strains are determined not only by their level of protease activity but also by the nature of their corresponding enzyme proteins. The most studied proteases from *E. histolytica* related strains are summarized in Table 2.

### 3.1. *Entamoeba moshkovskii* or *Entamoeba histolytica* Laredo Type

The *Entamoeba histolytica* Laredo strain was isolated 50 years ago from a resident of Laredo (USA) who suffered from diarrhea, weight loss, and epigastric pain [213]. Further molecular studies and biological features revealed that the *E. histolytica* Laredo strain is identical to *Entamoeba moshkovskii* [214], a species with a worldwide distribution that is considered a nonpathogenic free-living amoeba. Recently, some studies have reported the possibility that *E. moshkovskii* could be a pathogenic species with the capacity to infect humans or be associated with gastrointestinal symptoms [215].

*E. moshkovskii* is frequently found in regions where amoebiasis is highly prevalent. It has been isolated from wastewater, freshwater from rivers and lakes, brackish water, and human feces samples; this last finding would seem to suggest that *E. moshkovskii* could be pathogenic. Both the Laredo strain and *E. moshkovskii* grow at room temperature as well as at body temperature (37°C). They present trophozoite and cystic forms, and their size varies depending on the strain. In culture, trophozoites are able to complete cycles of division, encystation, and excystation. Usually one to four nuclei are observed, but there are forms with more nuclei, which tend to occupy a central position in the mature cysts [213, 215, 216].

 When observed by light microscopy, *E. moshkovskii* does not have morphological features that permit its distinction from *E. histolytica* and *E. dispar* [217]. *E. histolytica* can grow at temperatures ranging from 27 to 36.5°C, while *E. moshkovskii* can grow at temperatures from 4 to 40°C and in the presence of low amounts of nutrients that are not suitable for the growth of other *Entamoeba* species. *E. moshkovskii* can also adapt to extremely hypotonic cultures as it develops a contractile vacuole, a characteristic that is not present under normal growth conditions [215]. An assay was recently reported for the differential detection of *E. histolytica*, *E. dispar* and *E. moshkovskii*.  This method consists of a single PCR step and is highly sensitive and potentially quantitative. It is possible to detect 0.2 pg *E. histolytica* and 2 pg *E. dispar* or *E. moshkovskii *DNA [218].

 Homogenates of the Laredo strain or *E. moshkovskii *were tested against RBCs from different mammalian species. The homogenates had significant activity against hamster RBCs, but human RBCs were highly resistant to lysis [212]. A comparison of the acid and neutral protease activities of the Laredo strain with other, less virulent strains revealed that the Laredo strain had significantly less activity [25]. This strain was tested for its capacity to digest native radioactive type-I collagen gels and produce liver abscesses in newborn hamsters. Laredo strain did not show collagenolytic activity and failed to produce lesions. Thus, the susceptibility to invasive infection may depend on the characteristics of the extracellular components of host tissues and the potential virulence of the parasites [141]. Additionally, this amoeba was unable to produce secreted electron-dense granules, which are associated with an increase in collagenolytic activity in *E. histolytica* [137]. Amoebic proteases and host leukocytes were studied in a model of acute experimental amoebiasis that was produced by the intratesticular injection of axenic trophozoites in rats. The degree of inflammation and necrosis produced by *E. moshkovskii* was indistinguishable from the control without lesions, and this was correlated with the protease activity measured against azocasein. In this case, the protease patterns of *E. histolytica* type Laredo and *E. moshkovskii* were different because *E. histolytica* type Laredo displayed high protease activity and caused minimal tissue damage. 

 The current evidence proposes that the risk factors for acquiring infections by *E. moshkovskii* are similar to those described for *E. histolytica* and *E. dispar*. However, the capacity of *E. moshkovskii* to grow in the environment and adapt to adverse conditions suggests that the risk of infection is even greater than that for *E. histolytica* or *E. dispar* infection [213, 215, 218]. 

### 3.2. *Entamoeba dispar*

*E. dispar *is a nonpathogenic (commensal) amoeba with a morphology, genetic background, cell biology, and host range similar to that of *E. histolytica*. Together, *E. histolytica *and *E. dispar *infect approximately 10% of the world's population, but *E. dispar *is much more common. Studies of isoenzymatic patterns, antigenicity, and genetics have led the WHO to declare that *E. histolytica* and *E. dispar* should be classified as distinct species based on genetic differences in multiple genes [133, 219]. Recently, *E. dispar* was reported to present pathogenic behavior depending on the strain and culture conditions [220]. *E. histolytica* equivalent genes for virulence have been detected in *E. dispar*. The importance of CPs in the pathogenesis of invasive amoebiasis is indisputable; CPs are important virulence factors and the main proteolytic enzymes in *E. histolytica* [34, 133]. 

 In the beginning of the 1990s, studies of *E. dispar* strains grown in xenic cultures revealed important differences in the proteolytic activity on albumin and gelatin compared to *E. histolytica* axenic and xenic cultures [221]. As discussed earlier, within the total sequence of the *E. histolytica *genome, more than 40 genes encoding CPs have been identified [71, 73, 222]; only 8 of these CPs are moderately expressed in culture [73]. The majority of the protease activity detected in *E. histolytica *lysates is the result of the *ehcp1, ehcp2*, and *ehcp5 *genes.  *E. dispar *encodes 4 genes (*edcp1 *to *edcp4*) homologous to those of *E. histolytica* [73, 133]. Functional genes homologous to *ehcp1 *and *ehcp5* are absent in this nonpathogenic species [35, 92]. 

 The characterization of proteases in *E. dispar* has revealed important insights for the understanding of invasive amoebiasis [34, 133]. CP genes that are associated with virulence were thought to be expressed in *E. histolytica* but not *E. dispar*, such as *acp1 *(*ehcp3*) [77]; a related homologue with 95% identity was identified in *E. dispar* (*edcp3*) [84, 223]. The same phenomenon was observed for the *ehcp2 *gene,  which was subsequently detected in clinical strains of *E. dispar* [35, 73, 77]. The strategy was changed, and *E. histolytica* proteases were expressed in *E. dispar*; EhCP2 was overexpressed in *E. dispar* by episomal transfection, yielding almost the same amount of total CP activity as *E. histolytica*. EhCP2 overexpression produced increased monolayer destruction in cultured mammalian CHO cells but was unable to produce liver abscesses in an animal model. The same result was observed when *E. dispar* was modified to overexpress EhCP5, a CP that is not present in this amoeba, *in vitro* [71, 149]. EhCP5 activity increased almost 3.0-fold, leading to greater monolayer destruction [87]. 

 In an effort to characterize the molecular basis for the failure to express a CP5-analogous enzyme in *E. dispar*, the *ehcp5*-containing genomic regions from *E. histolytica *and *E. dispar* were compared. The gene corresponding to *ehcp5 *is present and the location is conserved in *E. dispar*, but the gene is highly degenerate and does not contain any open reading frame, suggesting that the gene has been nonfunctional for a considerable period of time during the evolution of the nonpathogenic amoeba species [87]. Degeneration of the *edcp5* gene constitutes a loss of a specific and functional accessory molecule important for processing CP5 [87, 92, 224]. Recent advances in DNA-mediated gene transfer in *E. histolytica *may help to prove or disprove this hypothesis. 

In comparison with *E. histolytica*,  the expression of CPs in *E. dispar* is lower (CP activity in *E. histolytica* is 10–1000-fold higher than in *E. dispar*), which could explain the differences in pathogenicity between these species [35, 73]. However, it is not clear if the ability of *E. histolytica* to invade is due to the synthesis of more CPs or because these enzymes differ in activity [75]. Thus, the systematic comparison of *E. histolytica* and *E. dispar* constitutes an important area of research to identify and analyze factors that may be important for amoebic pathogenicity.

 After phagocytosis, cytoplasmic (EhCP3) and membrane-associated (EhCP2) proteases are released into phagocytic vesicles in *E. histolytica* and *E. dispar* [75]. A comparative analysis of maturation of phagolysosomes by acidification, recruitment of hydrolases, and degradation of phagosomal content between the two species was performed; this sequence of events is crucial for the ability to ingest and kill microorganisms and host cells. Phagosomes of *E. histolytica* are much larger and contain a greater number of bacteria than those of *E. dispar *and have more efficient acidification. These differences reflect the nature of proton pumping across the phagosomal membrane and membrane trafficking, leading to phagosome maturation. Acidification is essential for the activation of hydrolytic enzymes and degradative proteins such as EhCP1, EhCP2, EhCP4, and EhCP5, phospholipases, and lysozyme. Degradation in *E. histolytica* phagosomes occurs more rapidly than in *E. dispar *phagosomes, in agreement with the previous findings that CP activity in *E. histolytica* is 10–1000-fold higher than in *E. dispar *and that specific inhibitors do not prevent degradation in *E. dispar* phagosomes, possibly because the CPs from *E. dispar* are relatively more resistant to inhibitors or these enzymes are not essential for digestion in phagosomes. These differences in phagosome degradation and the enzymes involved in host cell degradation could determine the difference in the outcome of infection with these two species [225].

Recently, the capacity of the *E. dispar *ICB-ADO strain to cause liver damage and destroy cell culture lines in the presence of common intestinal bacteria was reported. This amoeba, which was isolated from a patient who was symptomatic but not dysenteric, was able to induce liver necrosis in hamsters in an even more severe form than that produced by *E. histolytica* and exhibited high CP activity [220]. Thus, *E. dispar* could exhibit pathogenic behavior with the potential to have an invasive role, depending on the strain and host conditions. The authors also discussed whether *E. dispar* is a commensal or pathogenic parasite; they present evidence that bacteria are able to contribute to the regulation of factors such as proteases, which could have an important impact on *E. dispar* virulence.  Liver lesions with a large number of amoebae are in close contact with hepatocytes and induce an inflammatory reaction, ultimately resulting in an extensive necrotic area with a limited number of trophozoites compared to *E. histolytica* HM-1:IMSS. By contrast, the damage produced to MDCK cells was less extensive. Accordingly, the proteolytic activity of an *E. dispar* polyxenic strain was increased by almost 75% in comparison with a monoxenic strain and by 50% in comparison with *E. histolytica* [220]. 

Research on *E. histolytica *and *E. dispar* has primarily focused on the large family of CPs, which has been firmly linked to amoebic virulence, but numerous proteases are encoded in the *E. histolytica *genome. Only one copy of the gene is present in *E. dispar*; this gene is most closely related to *ehmsp-2*.  The *ehmsp-1* gene functions in the regulation of adherence and also affects motility, tissue culture monolayer destruction, and phagocytosis, as described previously [226]. 

### 3.3. *Entamoeba invadens*

*E. invadens* is a protozoan that causes invasive disease in reptiles and has the same two stages of life cycle and pathogenic potential as *E. histolytica*. The trophozoite is the stage that lives within the infected host, while the transmissible stage is the quadrinucleated cyst. In some of its hosts, *E. invadens* causes liver and intestinal damage with similar pathology to that of *E. histolytica*-infected individuals. This amoeba is studied as a model of encystation and excystation in the *Entamoeba* genera since *E. histolytica in vitro* encystation has not been achieved [227, 228]. Proteases and, in particular, CPs are important for several cellular processes such as differentiation or host cell invasion. In contrast to *E. histolytica*, very little is known about the *E. invadens* genome [227]. 

 To study proteases and their role in virulence in *Entamoeba *spp., amoebic cytopathogenicity was compared among several axenized strains. Each amoeba species possesses distinctive virulence, defined by the rate of tissue destruction, which, in turn, can be correlated with the presence or absence of hydrolases. To identify specific cytopathic effects, hemolytic profiles of homogenates of two *E. invadens* strains were compared in RBCs of different origins. The *E. invadens* strain PZ has no activity on RBCs, while the strain IP-1 has very high specific hemolytic activity against hamster and mouse RBCs [212]. A subsequent study revealed that the enzymatic activity is similar in *E. histolytica* and *E. invadens*. Proteases from these species were stable under denatured conditions and were inhibited efficiently by NEM and IA, indicating that they are CPs [229]. The importance of *E. invadens* CPs became apparent upon the observation that the major CP degrades azocasein at optimal pH of 4.8 and is similar to the cathepsin B-like CP of *E. histolytica*.  In addition, *E. invadens* proteases are activated by DTT and inhibited by typical CP inhibitors (E-64, IA, and PHMB). Further studies demonstrated that CPs may be critical for the survival of *E. invadens* as the specific inhibition of these proteases may ultimately interrupt parasite transmission [40]. Interestingly, in the presence of SDS, the *E. invadens* CPs seem to be processed for autoproteolysis [33].

 A model of acute experimental amoebiasis in rat testis was used to study the role of proteases. In this work, the severity of testicular lesions was correlated with the level of protease activity observed in *E. histolytica* strains and in *E. moshkovskii*. The exception was *E. invadens*, which displayed high protease activity but produced minimal testicular damage, suggesting that both the pathogenicity and degree of virulence of various amoebic strains are determined by the level of protease activity and by the type of enzyme [32]. Several proteases with homology to the *ehcp3 *and* ehcp2* genes of *E. histolytica* have since been reported in *E. invadens*. Purified *E. invadens *CPs exhibit substrate specificity similar to that of *E. histolytica* CPs and are inhibited by specific inhibitors. *E. invadens* possesses CPs, MPs, and SPs. CPs clearly play a role in encystation because the use of inhibitors reduces the degree of encystation, but this effect seems to be produced by decreased amoebic viability [38]. In addition to CPs, other proteins that are differentially expressed during encystation have been described, such as chitinases that are directed to the cell surface inside small secretory vesicles [230, 231].

 Proteases are involved in the differentiation process in *E. invadens*. The proteasome system is very important during the conversion of trophozoites into infectious cysts, as evidenced by the disruption of the encystation process by proteasome inhibitors, while CPs inhibitors only delay the process [232]. In the cyst, the most abundant proteases are serine-type proteases, which could be involved in the synthesis of chitin, most likely by activating the chitin-synthase proenzyme, because AEBSF, a specific inhibitor of SPs, prevented the formation of viable mature cysts. Thus, life cycle and transmission can be interrupted by specific inhibitors of SPs [39]. Therefore, both chitinase and SPs are essential for cyst wall destruction during the excystation process [37]. The participation of CPs in encystation and excystation of *E. invadens* was confirmed by using E-64 [36]. 

 Presently, several CP genes have been identified in *E. invadens* IP-1 based on their homology with genes in *E. histolytica* (*eicp-a3, -a5, -a9, -a11, -b7, -b9, -b10,* and *-c2*). Twenty of these genes are expressed during axenic parasite cultivation, whereas the remaining are not expressed or expressed at very low levels. The expression patterns of eight of the identified *E. invadens* CP genes were evaluated during the encystation or excystation processes. *eicp-b9* is the major gene expressed during encystation that is not involved in *Entamoeba* autophagy, but its specific function during encystation is unknown [227]. The MPs encoded by *ehmsp-1 *and *ehmsp-2* in *Leishmania major* were obtained in *E. histolytica *and* E. dispar* but were absent in *E. invadens*.  This implies that *ehmsp-1 *and *ehmsp-2* are the result of gene duplication just previous to the divergence of *E. histolytica *and *E. invadens*. Thus, *E. histolytica *is more closely related to* E. dispar *than to* E. invadens*,  but in some time,* E. dispar *had both gene copies before losing *edmsp-1* [226].

## 4. Pathogenic Free-Living Amoeba Species

Free-living amoebae are protist organisms distributed worldwide. A small number of species have been implicated in human disease: *Naegleria fowleri, Acanthamoeba *spp., and *Balamuthia mandrillaris*. Some of these infections are opportunistic and mainly occur in immunocompromised hosts, while others affect healthy people only under certain conditions. Although the number of infections caused by these protozoa is low, research is needed due to the difficulty in diagnosing these diseases and the lack of specific treatments, which results in high mortality, mainly by encephalitis. Unfortunately, molecular studies of free-living amoebae have not been performed. However, some virulence factors of these parasites have been purified and characterized. The most studied proteases from pathogenic free-living amoeba species are summarized in Table 3.

### 4.1. *Acanthamoeba* spp

*Acanthamoeba *is the most common free-living amoeba genus and is ubiquitously distributed. *Acanthamoeba *species are opportunistic amphizoic protozoa that are found in soil, air, and water, although they have also been isolated from vegetables and some animals. These amoebae cause several diseases in humans, such as granulomatous amoebic encephalitis (GAE) in immune-compromised patients and keratitis in contact lens wearers. Protozoa are resistant to diverse environments because they can tolerate a wide range of osmolarity, salinity, pH, and temperature. *Acanthamoeba* species that cause human infections are *A*.* castellanii, A. polyphaga*,* A. culbertsoni*,* A. hatchetti*,* A*.* healyi*,* A. astronyxis*, *A. lugdunensis* and* A*.* divionensis*. All of these species have a simple life cycle with two phases, a vegetative stage or trophozoite (8–40 *μ*m) and a resistant stage or cyst (8–29 *μ*m). The name *Acanthamoeba* comes from the presence of fine spine-like structures or acanthopodia projecting outward from the surface of the body [233].

 Typical forms of infection by *Acanthamoeba *spp. are GAE and nasopharyngeal or cutaneous invasion; amoebae spread by the hematogenous via. GAE is rather rare; approximately 150 cases have been described worldwide. Due to the difficulty in diagnosis, it is possible that other cases of GAE have been misdiagnosed. By contrast, more than 30 cases of keratitis due to *Acanthamoeba *were reported in Chicago (USA) alone. As of August 2006, more than 5000 cases of keratitis due to *Acanthamoeba* are estimated to have occurred in the United States, but the actual number of infections around the world is unknown. Large numbers of cases have also been reported in the United Kingdom and India [52].

 The adherence of *Acanthamoeba* trophozoites to target cells or tissues is an important step in host invasion. Amoebae can adhere to human and animal corneal epithelial cells. Omaña-Molina et al. [234] reported the early adhesion steps of *A. castellanii* and *A. polyphaga* trophozoites to hamster cornea. After adherence to the epithelial surface, the trophozoites form clumps and migrate to cell borders, causing separation of adjacent cells and cytopathic damage in distal cells. Amoebic adhesion may be mediated by a 130 kDa mannose-binding protein (MBP), which is a surface-expressed protein [235]. Other adhesins include a laminin-binding protein of 28 kDa [236] and a 55 kDa protein that binds to the laminin of the pathogenic strain *A. culbertsoni* [237]. Interestingly, *A. polyphaga *binds to the ECM proteins collagen type IV, laminin, and fibronectin [238], and calcium enhances this binding [239]. In these interactions, amoebae exhibited a strong attachment to the basal membrane components laminin and collagen IV. The adherence to these molecules leads to secondary responses such as phagocytosis and toxin production that result in host cell death via the phosphatidylinositol 3-kinase (PI3-K) pathway [240]. 

 In *Acanthamoeba*, several groups have shown that adherence to the corneal epithelium results in the production of diverse proteases [41, 241]. The action of the proteases includes damage to the collagen shield and degradation of glycoproteins such as plasminogen, fibrinogen, laminin, and hemoglobin [241]. In addition, *Acanthamoeba* species display plasminogen activator activity, which can trigger host matrix MPs, leading to degradation of basement membranes. In another study, Alizadeh et al. [241] demonstrated that the addition of mannosylated proteins to the cornea of Chinese hamsters induced the expression of a protease of 133 kDa (MIP133) that mediated apoptosis of corneal epithelial cells, facilitated corneal invasion, and degraded the corneal stroma. *Acanthamoeba *spp. also possess hydrolytic enzymes, such as elastases [242], phospho-lipases [243], SPs [47, 244–247], CPs [47, 244–247], and contact MPs [248].

 In the last twelve years, several proteases from distinct species of *A. polyphaga* have been purified and characterized. Proteases have been obtained from both total crude extracts and conditioned culture medium and tested with natural and synthetic substrates. Alfieri et al. [244] utilized gelatin-containing gels and azocasein to demonstrate azocasein hydrolysis by cell lysates at an optimal pH of 4.0-5.0; this hydrolysis was predominantly associated with CP activity. By contrast, culture SN contained significant azocasein hydrolyzing activity corresponding to SPs. We [47] performed a partial biochemical characterization of proteases in total crude extracts and conditioned culture medium from *A. castellanii *and *A. polyphaga *strains by using gelatin-containing gels and azocoll assays (both denatured type-I collagen). Interestingly, 17 proteolytic bands distributed between both *Acanthamoeba *strains were observed. The Mr of these bands ranged from 30 to 180 kDa in *A*. *castellanii *and 34 to 144 kDa in *A. polyphaga*. Incubation with protease inhibitors revealed that the proteolytic activities mainly belonged to the SP type, followed by CPs, in both total crude extracts and conditioned culture medium.

 Numerous proteases in *Acanthamoeba *are able to degrade ECM components. He [249] described the presence of a collagenolytic enzyme from *A*.* castellanii* culture medium that partially digested collagen shields after 4 h of incubation, with complete degradation by 8 h. Similar results were obtained with shields incubated in purified collagenase solutions. More importantly, when naïve Lewis rats were treated with *Acanthamoeba*-conditioned cultured medium, corneal lesions were produced that were clinically similar to those found in biopsy specimens of human patients diagnosed with acanthamoebic keratitis. The use of nonspecific protease inhibitors and EDTA-Na in the *Acanthamoeba*-cultured medium completely blocked the degradation of collagen shields, and the use of EDTA-Na *in vivo* also blocked amoebic collagenase activity. The authors demonstrated that the parasite-derived culture medium most likely contained considerable amounts of collagenase and low concentrations of other proteolytic enzymes. Thus, the authors speculated that, in the initial infection, *Acanthamoeba*-derived collagenase acts as a priming agent to produce collagenolysis, edema, and neutrophils infiltration in human keratitis.

 Excretory and secretory products of *A. polyphaga* culture have been described by [247], who observed major bands of proteolytic activity in nondenatured gelatin substrate gels with Mr of 36, 49, and 66 kDa in homogenate and excreted/secreted products. These proteases showed a wide pH range of activity, with an optimum at pHs 7–9. The authors also described the collagenolytic activity of *A. polyphaga* culture medium on the substrates azocoll and gelatin and native type-I collagen. They concluded that *A. polyphaga *secretes multiple SPs, CPs, and MPs and that all of these proteases contribute to the collagenolytic effect. Recently, Ferreira et al. [250] characterized elastase activities in the culture medium of *A. polyphaga*. These activities are in the range of 70–130 kDa, with an optimal pH of 7.5; in addition, these activities are inhibited by PMSF, antipain, chymostatin, and 1,10-phenanthroline and partially reduced by elastinal and EDTA. This study demonstrated that amoebic trophozoites secrete elastase activities and suggested high-molecular-weight SPs as possible elastase candidates.

 A secreted SP of *A. healyi* was purified by Kong et al. [251].  The protease has a molecular weight of 33 kDa and an optimum pH of 8.0; interestingly, the optimum temperature was 40°C. This protease degrades type-I and -IV collagen and fibronectin. The protease activity is inhibited by PMSF and DIFP, both SP inhibitors. Na [42] purified a secreted SP from *A*.* castellanii *with an approximate Mr of 12 kDa. This molecule is a chymotrypsin-like protease that can degrade various substrates, such as collagen, fibronectin, laminin, sIgA, IgG, plasminogen, fibrinogen, hemoglobin, and rabbit corneal protein. The purified protein was also used to test cytopathogenicity toward HEp2 cells, which resulted in a loss of viability within 12 h. The cytopathogenic events were completely inhibited when the protease was pretreated with PMSF before addition to the HEp2 cells.

 A 33 kDa SP secreted by *A*. *lugdunensis *was purified by Kim et al. [46]. The protease showed a pH optimum of 8.5 and a temperature optimum of 37°C. This protease is able to degrade collagen types I and IV, fibronectin, fibrinogen, hemoglobin, albumin, IgG, and IgA. PMSF inhibited nearly all of the protease activity. Also, the same authors [252] reported that this 33 kDa protease could be purified from other *Acanthamoeba *strains with different degrees of virulence.

 Sissons et al. [45] identified two proteases of 130 and 150 kDa from an *Acanthamoeba *isolate capable of inducing GAE. The 130 kDa protease was inhibited by PMSF, suggesting that it is an SP, whereas the 150 kDa protease was inhibited by 1,10-phenanthroline, suggesting that it is an MP. Both proteases exhibited maximal activity at neutral pH and over a range of temperatures. These proteases degrade ECM components such as collagen types I and III, elastin, and plasminogen, as well as casein and hemoglobin. Finally, [41] described the partial biochemical characterization of proteolytic enzymes secreted by *Acanthamoeba *spp. trophozoites isolated from the corneal tissues of different patients. Different enzymatic patterns of proteases were observed that varied between single and multiple protease activities. Low-molecular-weight SPs were secreted by trophozoites and were associated with a more severe clinical course of keratitis. Protein extracts of *Acanthamoeba *trophozoites were assayed for specific activity against type-I collagen. Collagenases were observed in amoebic extracts of different patients. These isolates produced a single band of approximately 36 kDa. All of these patients suffered from severe infections. Consequently, *Acanthamoeba *proteolytic enzymes could play a role in the degree of virulence and clinical manifestations of disease in human keratitis.

 Pathogenic *Acanthamoeba *species are also known to infect the central nervous system (CNS), resulting in fatal GAE disease. The pathophysiological complications of this disease include induction of proinflammatory responses, invasion of the blood-brain barrier and connective tissue, and neuronal damage. Intranasal, intrapulmonary, and intracardiac inoculations of trophozoites lead to invasion of the CNS, suggesting that amoebae can enter by different routes, including the blood-brain barrier [253]. Several lines of evidence suggest that *Acanthamoeba *entry into the CNS most likely occurs at the cerebral capillary endothelium, including the observation that lesions are more frequent in the brain parenchyma of GAE lesions [253–255]. Some studies have focused on *Acanthamoeba *interactions with primary human brain microvascular endothelial cells (HBMECs) and have demonstrated that amoebae produce HMBEC dysfunction [256]. Proteases appear to play an important role in weakened tight junctions in HBMECs cultures [257]. *Acanthamoeba *protease-mediated disruption of HBMECs can be inhibited by the SP-inhibitor PMSF, implicating SPs in blood-brain-barrier perturbation [45, 257]. Furthermore, recent studies have shown that *Acanthamoeba *proteases target ZO-1 and occludin proteins [258]. More studies are needed to comprehend the importance of *Acanthamoeba* spp. proteases in the diseases that caused by these species. 

### 4.2. *Naegleria* spp

*Naegleria *spp. are amoeboflagellates that are found worldwide in warm freshwater and that feed mostly on bacteria. These amoebae transform from trophozoites to the flagellate form if nutrients are limited in order to move toward other rich nutrient niches. Amoebae can also transform into cysts to survive adverse conditions. Species of *Naegleria *have been known for over a century [259], but it was only approximately 40 years ago that one species, called *Naegleria fowleri, *was found to cause primary amoebic meningoencephalitis (PAM) in humans [260]. There is a strong indication that the pathogenic *N. fowleri *evolved from the nonpathogenic species *N. lovaniensis* [259]. Only 235 PAM cases have been reported worldwide, so the disease is rare. PAM is an acute, fulminant, and often fatal disease that occurs mainly in apparently healthy children or young people with a recent history of swimming, among which only around 5% of patients survive [259]. Infection is acquired by exposure to water in ponds, pools, or lakes contaminated with *N. fowleri* [259, 260]. 

 Other researchers and our group have found that *N. fowleri *trophozoites gain access to the CNS by crossing the olfactory bulbs in experimental animals [261, 262]. Once in the CNS, amoebae divide rapidly causing inflammation associated with tissue destruction, leading to death in a few days. The mechanisms involved in the tissue invasion and destruction are poorly understood. However, various *in vitro* studies suggest the presence of numerous virulence factors. These factors include adhesins [263], pore-forming proteins [264, 265], phospholipases [266], contact-dependent lysis [267], elastase [242], and secreted proteolytic enzymes with cytopathic effects [48, 50]. 

 There are few reports concerning the adherence of *N. fowleri* to ECM proteins. An integrin-like molecule that binds to immobilized fibronectin has been reported [263]. This protein was described as an *α*-integrin subunit with a role in cytotoxicity. Shibayama et al. [268] described the interaction of *N. fowleri* with human type-I collagen. Recently, adhesion to collagen and fibronectin by the pathogenic strain *N. fowleri *and the nonpathogenic *N. lovaniensis* was compared,  revealing greater adherence of *N. fowleri *to fibronectin [269, 270]; *N. fowleri *presents higher levels of surface glycoconjugates that contain *α*-D-glucose and terminal *α*-L-fucose residues than *N. gruberi*. Cytosolic and membrane glycoconjugates were more highly expressed in *N. fowleri *than in *N. gruberi*. These differences could be related to the adherence to different substrates, and therefore they could also be related to the pathogenesis of *N. fowleri*.

 Aldape et al. [48] partially purified a secreted protease of 30 kDa consisting of two proteins from* N*. *fowleri*. The biochemical properties of the two forms of *N*. *fowleri *protease activity were indistinguishable, suggesting that they may be posttranslationally modified isoforms of the same gene product. This activity was abolished by E-64 and leupeptin, both CP inhibitors. Trophozoites or secreted protease activities were able to degrade mainly collagen and elastin; this effect was inhibited by Z-FA-FMK, a specific CP inhibitor. We have described proteolytic activities from *N. fowleri *and *N. gruberi *that are able to degrade azure and azocoll at 37°C. These activities were mainly inhibited by the CP inhibitors PHMB and E-64, which indicates that the main protease activities in *N. fowleri* are thiol-proteases, although there are also lesser quantities of SP activity [50]. More studies are needed to elucidate whether specific proteases from *N. fowleri *can degrade ECM proteins such as type-I and -IV collagen, fibronectin, elastin, and laminin.

 We recently evaluated the role of mucins in the natural immune response and the role of the mucinolytic activity of trophozoites of *N. fowleri* [49].  A CP with Mr of 37 kDa with mucinolytic activity was identified. This protease could be an important molecule in mucin degradation and evasion of the host innate immune response. The study of *Naegleria *virulence factors is still limited; therefore, much future work is needed, particularly with respect to the role of proteases in the invasion of the CNS. 

### 4.3. *Balamuthia mandrillaris*

*Balamuthia mandrillaris* is the causative agent of *Balamuthia *granulomatous amoebic encephalitis, a life-threatening brain infection [271]. First isolated from the brain tissue of a mandrill baboon [272], *B*. *mandrillaris *is naturally found in soil [273] and occasionally invades humans to cause an insidious infection that can occur in either immunocompetent or immunosuppressed patients. To date, there have been approximately 120 reported cases of *Balamuthia *encephalitis worldwide, with only two survivors [274]. The incubation period is long and usually results in drastic neurological damage that includes single and multiple space-occupying brain lesions. The amoebic entry routes may be the nasal mucosa or skin injuries resulting from traumas [275]. Studies in mouse models have shown that *B. mandrillaris *is able to migrate through the olfactory nerve pathway and access the brain in this form [276, 277]. Until now, there have been few *in vitro* studies in *B. mandrillaris*. This amoeba has cytotoxic activity against monkey kidney cells [278, 279] and HBMECs [280], but the cellular mechanisms of cytopathogenesis are unknown. Rocha-Azevedo et al. [281] described the specific binding of *B. mandrillaris *to three ECM proteins *in vitro*: collagen-I, fibronectin, and laminin-1. Binding of amoeba to laminin was greater than that to collagen and fibronectin. The authors found that binding was inhibited when the amoebae were pretreated with sialic acid.

 There is little information about protease activities in *B. mandrillaris*. Matin et al. [51] used isolates of *B. mandrillaris* (from human and baboon) to describe protease activities in zymographic assays that revealed major protease bands with approximate Mr of 40–50 kDa. The protease bands were inhibited by 1,10-phenantroline, suggesting that these activities belong to the MP group. These activities were observed over a pH range of 5–11, with maximum activity at neutral pH and 42°C. The *B*. *mandrillaris *proteases could degrade type-I and -III collagen, elastin, and plasminogen. The authors also demonstrated that these proteases do not participate in the cytotoxic effects on HBMECs. 

 A complete understanding of the pathogenic molecular mechanisms of this disease is needed and may lead to the identification of potential targets for therapeutic management and an accurate diagnosis.

## 5. Concluding Remarks

The occurrence of tissue damage during infection is a complex phenomenon that is mediated by virulence factors from the parasite as well as exacerbated responses from the host. The numbers of amoebic surface and secreted proteases suggest that they are intended not only for nutrient acquisition but also as virulence factors. It is well established that there is a correlation between virulence and the level of protease expression in virulent amoebae, such as *E. histolytica *or* Acanthamoeba*, but not in other species such as *E. invadens*, for which the quality and not the quantity of expression could be important. This could also be true for proteases of other species previously considered nonpathogenic amoebae, such as *E. dispar*. Parasite tissue destruction may require the participation of many types of protozoan parasite proteases: CPs, SPs, and MPs. The predominant type of protease varies depending on the species; for example, *E. histolytica* uses mostly CPs, while *Acanthamoeba* spp. prefers SPs. 

 There are a great variety of protease targets such as mucin, ECM components, tight junction proteins, immunoglobulins, complement, and cytokines. Iron-containing proteins can also be included as protease targets because iron scavenger capacity is currently considered a virulence factor for pathogens; pathogens must overcome host multiple strategies that limit iron availability as an innate host defense mechanism. In addition, some proteases have a role in adhesion or encystation, which are essential for establishing and transmitting infection. Thus, the lack of proteolytic function of some proteases demonstrates the multipurpose potential of these valuable virulence factors. 

 Amoebic proteases appear to be nonspecific because they degrade a wide variety of substrates *in vitro*; however, to correctly assess the role of each protease in infection, it is necessary to evaluate parasite protease activities with models, *in vitro* models of complex substrates such as 3D synthetic ECM, and *ex vivo* assays to test the proteolytic activity under conditions similar to that of an actual infection. The participation of host cells and bacteria in the invasion of parasites is also relevant because these cells can greatly influence the amoeba protease profile, as has been reported for *E. histolytica* and *E. dispar*, the protease profiles which change extensively after contact with host cells or bacteria. Although a lot of valuable information has been obtained studying axenically grown trophozoites, this information should be carefully extrapolated to what occurs during infection.

 Previously, it was thought that amoebic proteases were not able to exert cytopathic damage to cells; however, exciting new evidence suggests that, by degrading the anchorage points of host cells such as the ECM, *E. histolytica* proteases lead to a special type of apoptosis called anoikis in host cells. In addition, in *Acanthamoeba*, a protease has been described that mediates apoptosis of corneal epithelium cells. 

 For many of these parasites, the identities of the proteases are unknown because the reports refer only to proteolytic activities of certain molecular mass ranges, with the exception of the extensively studied* E. histolytica*. Therefore, it is important to identify each gene responsible for these proteolytic activities to obtain a better understanding of parasite pathogenesis. Once the gene has been identified, it is important to use parasites in which protease genes are deleted or overexpressed. Such studies will be of great value to elucidate the actual roles of parasite proteases as virulence factors. This type of study has only been performed in *E. histolytica* and *E. dispar*, with restrictions. In *E. histolytica,* the only available strategy requires the use of the G3 strain in which the amoebapore is silenced as a genetic background to perform the knockdown of the protease gene. This strategy has the limitation that a protease is assessed as a virulence factor with an already attenuated trophozoite. In addition, silencing strategies have proven to have some degree of nonspecificity, so it has been difficult to assign a function to a specific protease.

 Finally, most patients suffering from amoebiasis express antibodies to trophozoite CP, making these proteases an attractive potential vaccine target or a potential tool to improve the early diagnosis of human parasitic diseases. Because transmission, invasion, and life cycle could be interrupted by using specific parasite proteases inhibitors, the study of proteases and their specific inhibitors is relevant to the search for new therapeutic targets or to increase the power of drugs currently used to treat these diseases, such as in the reduction of tissue damage when specific inhibitors are used in animal models.

## Figures and Tables

**Table 1:** Cysteine proteases of *Entamoeba histolytica *that degrade host proteins and their implication in virulence.

Tissue or protein	Purified	Name	Mr (kDa)	Comment, localization	Reference
ECM components					

Collagen	Yes	EhCP1	27	*In vitro, *surface	[6]
	Yes	EhCP2	26	*In vitro*, membrane-associated	[7]
	Yes	EhCP112	35.5	*In vitro *(zymogram), surface	[8]
	Yes	EhCP5	30	*In vitro* (zymogram), and it is essential *ex vivo* for the cleavage of the collagen network, surface	[9, 10]
	Yes	—	56	*In vitro, *secreted	[11]
	No	—	72	*In vitro *(zymogram), crude extracts and EDG	[12]
	Yes	—	27–29	*In vitro *	[13]
Laminin	Yes	EhCP4	26	*In vitro*, secreted and nuclear	[14]
	Yes	—	56	*In vitro, *secreted	[11]
	Yes	—	27–29	*In vitro *	[13]
Fibronectin	Yes	EhCP112	35.5	*In vitro *(zymogram), surface	[8]
	Yes	—	56	*In vitro, *secreted	[11]
	Yes	—	27–29	*In vitro *	[13]
Gelatin	No	—	110, 68, 56, and 22	Induced by collagen, conditioned medium	[15]

Immunoglobulins					

IgA	Yes	EhCP4	26	*In vitro*, secreted and nuclear	[14]
	Yes	—	70	*In vitro *	[16]
IgG	Yes	EhCP5	30	*In vitro*, surface	[9]
	Yes	EhCP4	26	*In vitro*, secreted and nuclear	[14]
	Yes	EhCP1	27	*In vitro,* surface	[17]
	Yes	—	56	*In vitro, *secreted	[11]

Complement					

C3	Yes	EhCP4	26	*In vitro*, secreted and nuclear	[14]
	Yes	EhCP1	27	*In vitro, *surface	[17]
	Yes	—	56	*In vitro *	[18]
C3a and C5a	Yes	—	56	*In vitro *	[19]

Cytokines					

proIL-18	Yes	EhCP4	26	*In vitro*, secreted and nuclear	[14]
	Yes	EhCP5	30	*In vitro*, surface	[20]
	Yes	EhCP1	27	*In vitro, *surface	[17]
proIL-1*β*	Yes	EhCP1	27	*In vitro, *surface	[21, 22]
		EhCP2	26	*In vitro*, membrane-associated	[21, 22]

Iron-containing proteins					

Hemoglobin	Yes	EhCP112	35.5	*In vitro *(zymogram), surface	[8]
	Yes	EhCP5	30	*In vitro *(zymogram), surface	[9]
	Yes	—	41, 27	*In vitro, *intracellular	[23]
	No	—	32, 40	*In vitro,* total extract	[24]
	Yes	—	22	*In vitro *	[25]
	Yes	—	16	*In vitro *	[26]
	No	—	116, 82, 28, and 21	Human, porcine, and bovine Hb *in vitro* (zymogram)	[27]
Transferrin	No	—	130, 43, 20, and 6	Total extract *in vitro* (zymogram)	[28]
	No	—	130, 70, 50, 35, and 30	Conditioned medium *in vitro* (zymogram)	[28]
Lactoferrin	No	—	250, 100, 40, and 22	Total extract *in vitro* (zymogram)	[29]
Ferritin	No	—	100, 75, and 50	Total extract *in vitro* (zymogram)	[30]

Other proteins or tissues					

Mucin	Yes	EhCP5	30	*In vitro* (zymogram), although *ex vivo* is not needed to cross the mucus, surface	[9, 31]
Proteoglycan	Yes	EhCP2	26	*In vitro*, membrane-associated	[7]
Villin	Yes	EhCP1	27	*In vitro, *surface	[14]
	Yes	EhCP4	26	*Iin vitro*, secreted and nuclear	[14]
Fibrinogen	Yes	EhCP5	30	*In vitro *(zymogram), surface	[9]
BSA	Yes	EhCP5	30	*In vitro *(zymogram), surface	[9]

EDG: electron-dense granules.

**Table 2:** Enzymes from* E. histolytica* related species that degrade host proteins and their implication in virulence.

Parasite	Tissue or protein	Name	Mr (kDa)	Catalytic type	Comment	Reference
*E. moshkovskii *						

	ECM components	—	*≈*45	CP	Zymogram and activity of proteases secreted in azocasein	[32]
FIC strain	Gelatin	—	90	CP	Zymogram	[33]
Laredo strain	Gelatin	—	90	CP	Zymogram	[33]

*E. dispar *						

	ECM components	EdCP3	*≈*30	CP	Sequence homology with *E. histolytica.* Located in cytoplasmic granules	[34, 35]

*E. invadens *						

PZ strain	Gelatin		99, 90	CP	Zymogram	[33]
IP101 strain	Gelatin		99, 90, 45	CP	Zymogram	[33]
IP-1 strain	Gelatin		56, 58–66, 44–54, 43	CP	Zymogram	[36]
	Gelatin		45	SP	Zymogram	[37]
	Gelatin		130–230, 55, 35	CP, MP	Zymogram	[38]
	Gelatin		130–230, 60	CP, MP	Zymogram	[39]
	Azocasein		28	CP	Zymogram	[40]

**Table 3:** Enzymes from free-living amoebae^a^ that degrade host proteins and their implication in virulence.

Pathogen	Tissue or protein	Purified	Name	Mr (kDa)	Catalytic type	Localization	Reference
*Acanthamoeba *spp.	Elastin	No		70–130	SP	Secreted	[41]
	Collagen, fibronectin, and laminin	Yes		12	SP	Secreted	[42]
	Corneal stroma (*ex vivo*), Collagen I and IV	Yes	MIP-133	133	SP	Secreted	[43]

*Acanthamoeba castellanii *	Corneal stroma (*ex vivo*), Collagen	Yes	MIP-133	133	SP	Secreted	[44]
	Collagen I and III Elastin	No		130	SP	Secreted	[45]
	Collagen I and III	No		150	MMP	Secreted	[45]
	Collagen I and IV Fibronectin, hemoglobin, albumin, IgA, and IgG	Yes		33	SP	Secreted	[46]
	Gelatin	No		178, 144, 123, 110, 78, 75, 72, 34, 30	SP	Crude extracts	[47]
	Gelatin	No		188, 157, 53, 34	SP	Conditioned medium	[47]

*Acanthamoeba polyphaga *	Gelatin	No		144, 105, 78, 72, 45, 40, 34	SP	Crude extracts	[47]
	Gelatin	No		72, 62, 50, 34	SP	Conditioned medium	[47]

*Acanthamoeba healyi *	Collagen I and IV Fibronectin, hemoglobin, albumin, IgA, and IgG	Yes		33	SP	Secreted	[46]

*Acanthamoeba lugdunensis *	Collagen I and IV Fibronectin, hemoglobin, albumin, IgA, and IgG	Yes		33	SP	Secreted	[46]

*Naegleria fowleri *	Collagen I and, elastin	Yes		30	CP	Secreted and cytoplasmic	[48]
	Bovine mucin	No		37	CP	Crude extracts	[49]
	Gelatin	No		130, 100, 73, 62	CP	Crude extracts	[50]
	Gelatin	No		310, 178, 164, 147	CP	Conditioned medium	[50]

*Balamuthia mandrillaris *	Collagen I and III, elastin, and plasminogen	No		40–50	MMP	Crude extracts	[51]
	Collagen I and III, elastin, and plasminogen	No		73 40–50	MMP	Conditioned medium	[51]

^a^All results have been performed in *in vitro* assays. MIP: mannose induced protease; SP: serine protease; CP: cysteine protease; MMP: matrix metalloprotease.

## Abbreviations of Inhibitors and Activators of Proteases

AEBSF: 4-(2-Aminoethyl) benzenesulfonyl fluoride
DIFP: Diisopropyl fluorophosphates
DTT: Dithiothreitol
EDTA: Ethylenediaminetetraacetic acid
E64: Trans-epoxysuccinyl-L-leucylamido-(4-guanidino)butane
IA: Iodoacetamide
NEM: N-ethylmaleimide
PHMB: p-Hydroxymercuribenzoate
PMSF: Phenylmethylsulfonylfluoride
TLCK: Tosyl-lysyl-chloromethylketone
TPCK: 1-Chloro-3-tosylamido-4-phenyl-2-butanone
Z-FA-FMK: Benzyloxycarbonyl-Phe-Ala-fluoromethyl ketone.
